# Reciprocal control of obesity and anxiety–depressive disorder via a GABA and serotonin neural circuit

**DOI:** 10.1038/s41380-021-01053-w

**Published:** 2021-03-26

**Authors:** Guobin Xia, Yong Han, Fantao Meng, Yanlin He, Dollada Srisai, Monica Farias, Minghao Dang, Richard D. Palmiter, Yong Xu, Qi Wu

**Affiliations:** 1 USDA/ARS Children’s Nutrition Research Center, Department of Pediatrics, Baylor College of Medicine, Houston, TX USA; 2 Department of Molecular Physiology & Biophysics, Vanderbilt University School of Medicine, Nashville, TN USA; 3 Department of Genomic Medicine, University of Texas MD Anderson Cancer Center, Houston, TX USA; 4 Departments of Biochemistry and Genome Sciences, University of Washington, Seattle, WA USA; 5 Howard Hughes Medical Institute, University of Washington, Seattle, WA USA; 6 Department of Molecular and Cellular Biology, Baylor College of Medicine, Houston, TX USA; 7 Present Address: Pennington Biomedical Research Center, Brain Glycemic and Metabolism Control Department, Louisiana State University, Baton Rouge, LA USA

**Keywords:** Neuroscience, Depression

## Abstract

The high comorbidity between obesity and mental disorders, such as depression and anxiety, often exacerbates metabolic and neurological symptoms significantly. However, neural mechanisms that underlie reciprocal control of feeding and mental states are largely elusive. Here we report that melanocortin 4 receptor (MC4R) neurons located in the dorsal bed nucleus of the stria terminus (dBNST) engage in the regulation of mentally associated weight gain by receiving GABAergic projections from hypothalamic AgRP neurons onto α5-containing GABA_A_ receptors and serotonergic afferents onto 5-HT_3_ receptors. Chronic treatment with a high-fat diet (HFD) significantly blunts the hyperexcitability of AgRP neurons in response to not only hunger but also anxiety and depression-like stimuli. Such HFD-mediated desensitization reduces GABAergic outputs from AgRP neurons to downstream MC4R^dBNST^ neurons, resulting in severe mental dysregulation. Genetic enhancement of the GABA_A_R-α5 or suppression of the 5-HT_3_R within the MC4R^dBNST^ neurons not only abolishes HFD-induced anxiety and depression but also robustly reduces body weight by suppression of food intake. To gain further translational insights, we revealed that combined treatment of zonisamide (enhancing the GABA_A_R-α5 signaling) and granisetron (a selective 5-HT_3_R antagonist) alleviates mental dysfunction and yields a robust reversal of diet-induced obesity by reducing total calorie intake and altering food preference towards a healthy low-fat diet. Our results unveil a neural mechanism for reciprocal control of appetite and mental states, which culminates in a novel zonisamide-granisetron cocktail therapy for potential tackling the psychosis-obesity comorbidity.

## Introduction

Obesity is a growing epidemic and is emerging as one of the leading health concerns over the recent two decades, with an estimate of nearly 1 in 2 adults in the USA to be developing obesity by 2030 [1–3]. Meanwhile, mental deficiency is among the most common behavioral traits in obese patients and genetic animal models predisposed to obesity [2, 4]. It has been reported that 43% of adults with depression are obese, and that adults who suffered from mental illnesses are more likely to be obese than those who are mentally healthy [4]. Although peripheral inflammation, hormonal dysregulation, and genetic deficiency is proposed to play a role in the comorbidity of obesity and mental disorders, the neural circuit mechanism and associated key neurotransmitter pathways are poorly understood [5–9].

AgRP neurons in the hypothalamus play a fundamental role in the control of appetite and body weight by sending broad projections to intra-and extra-hypothalamic targets including the paraventricular hypothalamic nucleus (PVH), lateral hypothalamic area (LH), paraventricular thalamic nucleus (PVT), and parabrachial nucleus (PBN) [10–19]. Meanwhile, emerging evidence shows that AgRP neurons engage in the control of diverse physiological processes including circadian rhythm, fertility, and pain sensation [20–22]. It is noteworthy that AgRP neurons modulate stereotypic responses during abnormal nutrient states [13, 23]. AgRP neurons also contribute to fasting-induced anxiolytic effects [24]. Together, these recent studies support a notion that a unique AgRP neural circuit may integrate peripheral hormonal signals with a category of metabolism-sensitive mental functions.

It has been established that GABA co-released from AgRP neurons promotes feeding and weight gain by the integration of various nutritional, hormonal, and neuronal signals [25, 26]. A class of benzodiazepine agonists, which acts through a specific binding site on GABA_A_ receptors, induces hyperphagia by enhancing food and taste palatability in every mammalian species studied [27]. Acute ablation of AgRP neurons or genetic inactivation of GABA signaling of young adult mice resulted in severe loss of body weight and abnormal glucose metabolism [28–30]. Pharmacological manipulation of AgRP postsynaptic targets within the PBN with a GABA_A_ receptor partial agonist, bretazenil, prevented starvation due to AgRP-neuron ablation, indicating that GABA_A_R signaling is vital for regulation of AgRP neurons [17].

The bed nucleus of the stria terminalis (BNST) acts as a hub for emotional and psychological regulation by making broad, hierarchical, connections with numerous brain regions [31]. The literature suggests that various subpopulations of intermingled neurons in the BNST are implicated to regulate neuroendocrine and behavioral processes through distinct GABA_A_ and 5-HTR family receptors [31–35]. For example, genetic disruption of GABA_A_-α1 signaling within the BNST corticotropin-releasing factor CRF)-expressing neurons increased anxiety-like behavior, coincident with an increase in plasma corticosterone concentration [36]. Treatment of GABA_A_ receptor agonist, muscimol or antagonist, picrotoxin within the BNST alleviates or enhances anxiety and depression responses, respectively [33]. Further, picrotoxin can block inhibitory postsynaptic currents of a subgroup of BNST PKC-δ neurons that inhibit feeding [37]. It is reported that multiple 5-HT receptor subtype mRNAs were expressed in BNST with correlation to mental states [38–40]. Although peripheral 5-HT_3_R signaling is reported to mediate obesity in mice [41–43], the central serotoninergic signaling pathways that engage comorbidity of obesity and emotional deficiency are largely unknown.

In this report, we found that the dorsal BNST (dBNST) plays a pivotal role in a reciprocal control of obesity and mood disorder comorbidity. GABAergic projections from AgRP neurons to the melanocortin 4 receptor (MC4R) neurons located in the dBNST modulates high-fat diet (HFD)-induced anxiety and depression-like responses. Genetic enhancement of the GABA_A_R-α5 signaling or suppression of the 5-HT_3_R signaling in the dBNST MC4R neurons not only abolishes mental dysfunction, but also robustly reduces food intake and body weight. More surprisingly, we demonstrate that a cocktail of zonisamide and granisetron by targeting GABA_A_R-α5 and 5-HT_3_R pathways, respectively, restores mental normality and promotes weight loss via altering food preference towards a healthy low-fat diet.

## Results

### HFD-induced anxiety and depression correlates with the desensitization of AgRP neurons

**Fig. 1:**
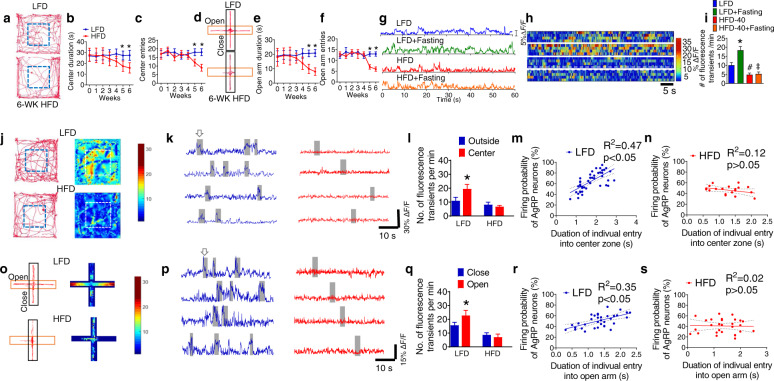
HFD-induced anxiety and depression correlate with the desensitization of AgRP neurons. **a** Representative activity tracers in OFT by WT mice fed with chow and 6 week of HFD. **b** Time spent in the center zone of the open field. **c** Entries of entering the center zone of the open field by mice fed with chow and HFD for 6 week. **d** Representative activity tracers in EPM in WT mice fed with chow and 6 week of and HFD. **e** Time spent in the open arm. **f** Entries of mice entered the open arm. Error bars represent mean ± SEM. *n* = 9–12 per group; *p < 0.05, LFD vs HFD at week 5 and 6, two-way ANOVA and followed by Bonferroni comparisons test. **g** Representative in vivo fluorescence traces of AgRP neurons in *Agrp*^*Cre*^ mice with injection of *AAV9-FLEX-GCaMP6f* into the ARC after chronic treatment of HFD or low-fat diet (LFD) under fed ad-lib or fasted condition. **h** Heat map of color-coded changes of fluorescence intensity in mice as described in (**g**). **i** The numbers of fluorescence transients detected in AgRP neurons in mice as described in (**g**). Error bars represent mean ± SEM. *n* = 10 per group; *p < 0.05, LFD vs LFD + Fasting; ^#^*p* < 0.05, LFD vs HFD; ^‡^*p* > 0.05, HFD vs HFD + Fasting; one-way ANOVA and followed by Tukey comparisons test. **j** Representative physical activity tracers (left) and heating maps of real-time AgRP neuronal activities (right) in OFT test in *Agrp*^*Cre*^ mice injected with *AAV9-FLEX-GCaMP6f* into the ARC followed with a chronic treatment of HFD or LFD under well-fed condition. Gray bars denote when the mice were within the center zone. **k** Representative in vivo fluorescence traces of AgRP neurons during OFT behavior test. **l** The number of fluorescence transients detected in AgRP neurons. **m** Correlation between the probability of AgRP neurons firing and the duration of the LFD-treated mice in the center zone of OFT. **n** Correlation between the probability of AgRP neurons firing and the duration of HFD-treated mice in the center zone of OFT. **o** Representative activity tracers (left) and heating maps (right) in EPM test in *Agrp*^*Cre*^ mice injected with *AAV9-FLEX-GCaMP6f* into the ARC followed with a chronic treatment of HFD or control low-fat diet (LFD) under well-fed and fasting condition. Gray bars denote when the mice were in the open arm. **p** Examples of in vivo fluorescence changes of AgRP neurons during EPM behavior test. **q** The number of fluorescence transients detected in AgRP neurons. **r** Correlation between the probability of AgRP neurons firing and the duration of the LFD fed mice in the open arm of EPM. **s** Correlation between the probability of AgRP neurons firing and the duration of HFD-fed mice in the open arm of EPM. Error bars represent mean ± SEM. *n* = 10 per group; *p < 0.05, one-way ANOVA and followed by Tukey comparisons test.

Numerous studies showed that diet-induced obesity is associated with anxiety/depression-like responses [6, 8, 44]. To examine the comorbidity of obesity and associated behavioral phenotypes in an animal model, 8–12-week-old male and female wild-type (WT) mice were fed with a 40% HFD or standard low-fat diet (LFD) for 6 weeks. At the end of the 6-week treatment, mice fed with HDF exhibited a small but significant increase in body weight in comparison to LFD-fed group (extended data Fig. 1a, b). To assess the anxiety and depression responses, we employed several behavioral assays, including the open-field test (OFT), elevated-plus-maze test (EPM), tail-suspension test (TST), forced-swim test (FST), and marble-burying test (MBT) in the daytime [45–47]. Echoing recent findings that HFD leads to rhythmic changes in locomotor activity during the nighttime but not daytime [21, 48], our behavioral assays performed in daytime were observed with no significant difference in total locomotion between LFD and HFD groups (extended data Fig. 1c–f). However, HFD-fed mice spent significantly less time in the center zone in the OFT compared to the LFD group (Fig. 1a–c). In addition, chronic HFD treatment induced a significant decrease in both duration and entries of open arm in the EPM test (Fig. 1d–f). Moreover, HFD-fed mice showed profound depression-like responses in the FST, TST, and MBT (extended data Fig. 1g–i). All of these results suggested that anxiety/depression-like responses developed along with chronic HFD treatment [6, 8, 44].

We utilized fiber photometry in vivo to assess whether chronic treatment of HFD plays a role in AgRP neuronal activity under various metabolic conditions (extended data Fig. 1j–l). In LFD-treated mice, overnight fasting caused hyperexcitability of AgRP neurons which was indicated by virally transduced calcium sensors (Fig. 1g–i) [49–51]. On the other hand, chronic exposure to HFD for 5 weeks not only reduced the basal activity of AgRP neurons but also abolished the fasting-induced hyperexcitability of AgRP neurons (Fig. 1g–i), indicating that HFD treatment desensitizes AgRP neurons in response to hunger signals. We further examined the activity level of AgRP neurons in the context of real-time physical locations during the OFT and EPM assays after chronic treatment of HFD [51]. Control animals when located in the center zone of OFT (denote in the gray boxes) exhibited hyperactivity of AgRP neurons (Fig. 1j–l). In contrast, HFD-treated mice had reduced AgRP hyperexcitability, correlating with the reduced duration and entries in the center (Fig. 1j–l). Statistical analyses suggested that the probability of AgRP neuronal hyperexcitability in LFD-treated mice is highly correlated with the duration of individual entry into the center zone, whereas such correlation is drastically diminished in HFD-treated mice (Fig. 1m, n). Similar to OFT results, the control mice in EPM showed significantly enhanced AgRP hyperactivity when entering the open arm (denote in the gray boxes) (Fig. 1o–q). We found a positive correlation between the duration of individual entry into the open arm and the hyperactivity of AgRP neurons (Fig. 1r). In contrast, chronic HFD significantly blunted AgRP hyperactivity and disrupted its correlation with the time of each appearance in the open arms (Fig. 1s). Collectively, our results suggest that the anxiety-depressive responses are mediated by HFD-induced desensitization of AgRP neurons.

### GABAergic AgRP neurons modulate anxiety and depression by projection to the dBNST

**Fig. 2:**
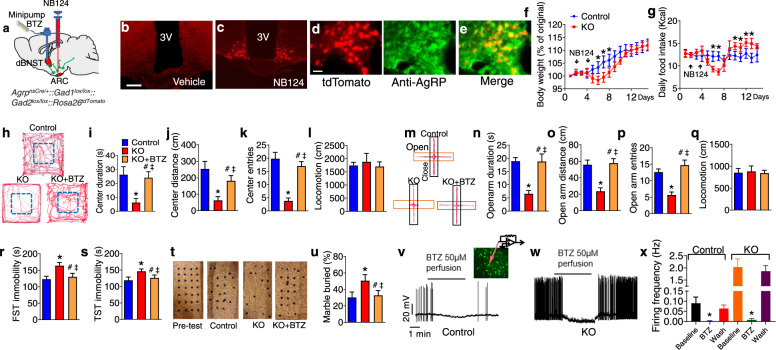
GABAergic AgRP neurons modulate anxiety and depression by projection to the dBNST. **a** Schematic image showing abrupt inactivation of GABAergic efferent of AgRP neurons followed by facilitating GABA signaling within the dBNST. **b**, **c** Expression of tdTomato (red) in *Agrp*^*nsCre/+*^*::Gad1*^*lox/lox*^*::Gad2*^*lox/lox*^*::*Ai14 mice with vehicle (**b**, control mice) and NB124 (**c**, KO mice) injection into the third ventricle. Scale bars in (**b**) for (**b**, **c**), 300 μm. **d**, **e** Co-localization of tdTomato and anti-AgRP in ARC after treating *Agrp*^*nsCre/+*^*::Gad1*^*lox/lox*^*::Gad2*^*lox/lox*^*::*Ai14 mice with NB124. Scale bar in (**d**) for (**d**, **e**), 100 μm. **f**, **g** Body weight (**f**), and daily food intake (**g**) by the control and KO mice. **h** Representative activity tracers in OFT by mice as described in (**b**) and (**c**) with a chronic infusion of either vehicle or BTZ (1 mg/ml) into the dBNST. **l**–**o** Time spent in the center zone (**l**), distance traveled in the center zone (**m**), the entries into the center zone (**n**), and total distance traveled in OFT (**o**). **p** Representative activity tracers in EPM by mice as described in (**h**). **q**–**t** Time spent in the open arm (**q**), distance traveled in the open arm (**r**), the entries into the open arm (**s**), and total distance traveled in OFT (**t**). **u**, **v** Immobility time in FST (**u**) and TST (**v**) by mice as described in (**h**). **w** Representative images of the MBT by mice as described in (**h**). **x** Percentage of the number of buried marbles in the MBT. Error bars represent mean ± SEM. *n* = 7–14; *p < 0.05, Control vs KO; one-way ANOVA and followed by Tukey comparisons test. **h** Firing activity of zsGreen-labeled dBNST neurons before and after treatment with BTZ (50 µM) in *Agrp*^*Cre/+*^*::*Ai14 mice with an injection of *AAV-DIO-WGA-zsGreen* into the ARC. **i** Firing activity of zsGreen-labeled dBNST neurons before and after treatment with BTZ (50 µM) in *Agrp*^*nsCre/+*^*::Gad1*^*lox/lox*^*::Gad2*^*lox/lox*^*::*Ai14 mice with an injection of NB124 and *AAV-DIO-WGA:ZsGreen* into the ARC. **j** Firing frequency of the ZsGreen-labeled dBNST neurons described in (**H** and **I**). Error bars represent mean ± SEM. *n* = 11–20 per group; *p < 0.05, Baseline vs BTZ in control mice; and *p < 0.05, Baseline vs BTZ in KO mice; one-way ANOVA and followed by Tukey comparisons test.

To identify the role of AgRP neurons and key neurotransmitter signaling molecules that contribute to mental functions, we take advantage of a newly established inducible genetic method to abruptly inactivate GABA biosynthesis in AgRP neurons [29]. Immunohistochemistry and qPCR results showed that central administration of NB124, a nonsense-codon suppressor, into the third ventricle of *Agrp*^*nsCre*^*::Gad1*^*lox/lox*^*::Gad2*^*lox/lox*^*::*Ai14 mice (AgRP^GABA-KO^ mice) resulted in expression of full-length, functional, Cre recombinase thereby effectively inactivating GABAergic signaling in AgRP neurons 4 days after drug treatment (Fig. 2a–e and extended data Fig. 2a) [29]. In line with our previous findings, AgRP^GABA-KO^ mice showed a transient but significant reduction of feeding and body weight after acute deletion of GABA signaling from AgRP neurons (Fig. 2f, g). Further, AgRP^GABA-KO^ mice exhibited a significantly enhanced anxiety-like responses in both the OFT and EPM assays (Fig. 2h–q). Moreover, AgRP^GABA-KO^ mice showed profound depression-like responses in the FST, TST, and MBT assays (Fig. 2r–u). To test whether loss of GABAergic inputs to the dBNST contributes to the anxiety-depressive phenotypes, BTZ was infused into the dBNST of AgRP^GABA-KO^ mice for 14 days. Importantly, these mental deficits were almost completely prevented by the chronic infusion of BTZ into the dBNST (Fig. 2h–u and extended data Fig. 3a–k). Results from the HPLC-MS assay indicated that the concentration of BTZ at nearby locations including the vBNST, LSv, IPACM, and CSF from the LV was significantly lower than that within the targeted dBNST (extended data Fig. 4a, b). To better understand the connectivity of AgRP neurons and dBNST, injection of ZsGreen-tagged wheat germ agglutinin (WGA), a Cre-dependent trans synaptic tracer, allows specific transduction into AgRP neurons [52]. Our in vitro electrophysiological recording results suggested that AgRP neurons establish monosynaptic connection with the downstream MC4R^dBNST^ neurons by releasing AgRP in the suppression of the postsynaptic MC4R signaling (extended data Fig. 5a–e). Furthermore, results derived from in vitro electrophysiological recording within the dBNST also showed that inactivation of GABA signaling from AgRP neurons caused robust hyperactivity of post-synaptic dBNST neurons, which in turn was abrogated by pre-treatment with BTZ (Fig. 2v–x and extended data Fig. 2b–c). These data reveal an unexpected role of GABAergic signaling from AgRP neurons into the dBNST in the regulation of mental states.

### An ARC^AgRP^→dBNST^MC4R^ circuit mediates anxiety, depression, and feeding

**Fig. 3:**
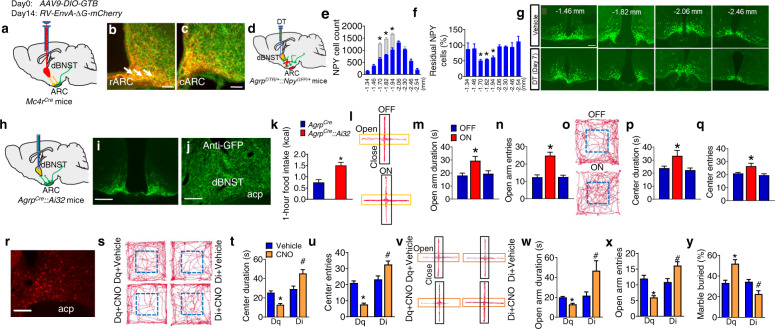
The ARC^AgRP^-dBNST^MC4R^ circuit mediates metabolism-associated anxiety and depression and feeding of HFD. **a** Schematic image of retrograde labeling of AgRP^ARC→dBNST^ neurons by injecting *AAV9-DIO-GTB* and *RV-EnvA-∆G-mCherry* viruses into the dBNST of *Mc4r*^*Cre*^ mice. **b**, **c** Overlay of rabies-mCherry and anti-AgRP within the rostral (**b**) and caudal (**c**) ARC in mice as described in (**a**). Scale bar in (**b**), 50 µm; scale bar in (**c**), 50 µm. **d** Diagram showing ablation of the BNST-projecting AgRP neurons via bilateral injection of DT or vehicle into the dBNST of *Npy*^*GFP/+*^*::Agrp*^*DTR/+*^ mice. **e**, **f** Number (**e**) and ratio (**f**) of NPY neurons in mice as described in (**e**). **g** Representative images showing AgRP neurons (green) in mice as described in (**e**). Data represent the mean ± SEM, *n* = 6–8 per group; *p < 0.05; one-way ANOVA and followed by Tukey post hoc test. **h** Photostimulation of AgRP axonal terminals within the dBNST of *Agrp*^*Cre*^*::*Ai32 mice. **i**, **j** Immunostaining of anti-GFP (green) within the ARC (**i**) and dBNST (**J**) of *Agrp*^*Cre*^*::*Ai32 mice. Scale bar in (**i**), 400 μm; scale bar in (**j**), 150 μm. **k** 1-h food intake of *Agrp*^*Cre*^*::*Ai32 mice upon photostimulation in the dBNST for 10 min at 20 Hz. **l**–**q** EPT (**l**–**n**) and OFT (**o**–**q**) test of *Agrp*^*Cre*^*::*Ai32 mice upon photostimulation in the dBNST. Error bars represent mean ± SEM. *n* = 7 per group; *p < 0.05; two-way ANOVA and followed by Bonferroni comparisons test. **r** Viral transduction pattern of *Mc4r*^*Cre*^ mice with an injection of *AAV9-FLEX-hM3Dq-mCherry* into the dBNST. Scale bar in (**r**), 150 μm. **s**–**y** EPM (**s**–**u**), OFT (**v**–**x**), and MBT (**y**) by the mice as described in (**r**) upon CNO treatment. Error bars represent mean ± SEM. *n* = 8–10 per group; *p < 0.05; two-way ANOVA and followed by Bonferroni comparisons test.

To further establish the connectivity between AgRP neurons and the dBNST, we performed a Cre-dependent, rabies-based, retrograde mapping of the AgRP→dBNST neural circuit [53]. *AAV9-DIO-GTB* virus was injected into the dBNST of *Mc4r*^*Cre*^ mice to allow specific expression of GFP-TVA-B19-glycoprotein in MC4R^dBNST^ neurons (Fig. 3a), followed by injections *of EnvA-G-deleted Rabies-mCherry* into the dBNST. Application of this retrograding strategy, we observed that neurons within the ARC that project to the MC4R^dBNST^ neurons comprised of a cohort of AgRP neurons (overlay as yellow) and other neuronal populations (as mCherry) that likely include a subset of POMC neurons (Fig. 3b, c and extended data Fig. 6a–c). The mapping results revealed that dBNST-projecting AgRP neurons were predominantly situated in the rostral ARC (Fig. 3b, c). We performed another profiling experiment by selective ablation of NPY/AgRP neurons that project to the dBNST in *Agrp*^*DTR*^*::Npy*^*GFP*^ mice with DT administration into the dBNST (Fig. 3d and extended data Fig. 8). Consistent with the retrograde tracing study, a significant loss of GFP-labeled NPY neurons was observed in the rostral ARC (Fig. 3e–g). Together, these results indicate that AgRP neurons located in the rostral ARC send long-range projections to MC4R^dBNST^ neurons. While the CRF^dBNST^ neurons are involved in the regulation of anxious behaviors [34, 38], histological results indicated that MC4R^dBNST^ neurons are a neuronal population distinct from the CRF^dBNST^ neurons (extended data Fig. 6d–i). Further, no overlapping profile was observed between the MC4R signaling and a group of BNST PKC-δ neurons recently reported to regulate inflammation-associated modulation of feeding (extended data Fig. 9a–f) [37].

In line with previous circuit mapping and functional studies, we found that photostimulation of channelrhodopsin (ChR2) expressed in AgRP-axonal terminals within the dBNST of *Agrp*^*cre*^*::*Ai32 mice promoted acute feeding (Fig. 3h–k) [11, 16, 54]. Moreover, photostimulation of AgRP^ARC-dBNST^ neurons caused a significant anxiolytic phenotype as revealed by OPT and EPM test (Fig. 3l–q and extended data Fig. 6j–m). In addition, chemogenetic stimulation of MC4R^dBNST^ neurons led to profound anxiety and depression-like phenotypes, while inhibition produced a robust anxiolytic and anti-depressant performance in all the behavioral paradigms. (Fig. 3r–y, extended data Fig. 6n–t, and extended data Fig. 7). These data demonstrate that the ARC^AgRP^-dBNST^MC4R^ circuit plays a vital role in appetite-induced mental dysfunctions and chronic feeding.

### GABA_A_-α5 within the MC4R^dBNST^ neurons regulates anxiety-depressive disorders and obesity

**Fig. 4:**
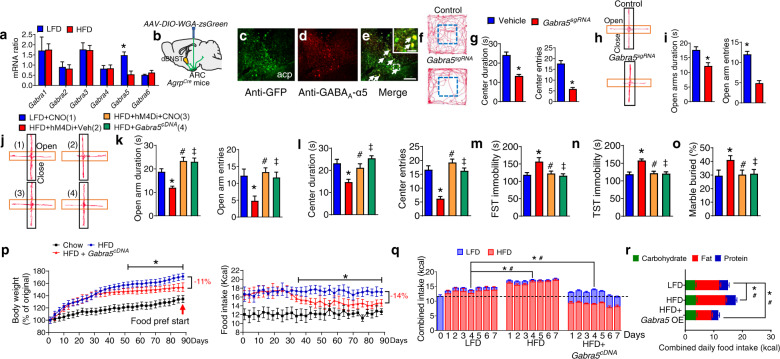
GABA_A_-α5 within the MC4R^dBNST^ neurons mediates HFD-induced mental dysfunction and obesity. **a** Expression level of major GABA_A_R-α5 subunits within the dBNST of WT mice treated with LFD or HFD Error bars represent mean ± SEM. *n* = 9 per group; *p < 0.05; two-way ANOVA and followed by Bonferroni comparisons test. **b** Diagram showing the trans-synaptic tracing by injection of *AAV9-FLEX-WGA-ZsGreen* into the ARC of *Agrp*^*Cre*^ mice. **c**–**e** Co-localization of the dBNST neurons directly innervated by AgRP neurons (**c**) and GABA_A_R-α5 (**e**). Inset in (**e**) is a magnified view of the boxed area showing colocalized neurons with arrow heads. Scale bar in (**e**) for (**c**–**e**), 150 μm; scale bar in the inset of (**e**), 30 μm. **f**–**i** OFT (**f**, **g**), and EPM test (**h**, **i**) by *Mc4r*^*Cre*^*::Rosa26*^*Cas9*^ mice with bilateral injection of either vehicle (Vehicle), *AAV9-FLEX-Gabra5*^*sgRNA*^*-tdTomato* (Gabra5^sgRNA^) into the dBNST. Error bars represent mean ± SEM. *n* = 7–10 per group; *p < 0.05; one-way ANOVA and followed by Tukey comparisons test. **j**–**o** EPM (**j**, **k**), OFT (**l**), FST (**m**), TST (**n**), and MBT (**o**) test in CNO or vehicle-treated *Mc4r*^*Cre*^ mice with either *AAV9-FLEX-hM4Di-mCherry* or *AAV9-FLEX-Gabra5*^*cDNA*^-*tdTomato* into the dBNST followed with a chronic treatment of HFD or LFD. Error bars represent mean ± SEM. *n* = 8 per group; *p < 0.05, LFD + CNO vs HFD + hM4Di+Veh; ^#^*p* > 0.05, LFD + CNO vs HFD + hM4Di+CNO; ^‡^*p* > 0.05, LFD + CNO vs HFD + *Gabra5*^*cDNA*^; one-way ANOVA and followed by Tukey comparisons test. **p** Body weight (left) and daily food intake (right) in LFD or HFD-treated *Mc4r*^*Cre*^ mice with injection of *AAV9-FLEX-Gabra5*^*cDNA*^-*tdTomato* into the dBNST. Error bars represent mean ± SEM. *n* = 9 per group; *p < 0.05, HFD vs HFD + Gabra5^sgRNA^; two-way ANOVA and followed by Bonferroni comparisons test. **q** Combined intake of HFD and LFD in a food preference test in mice as described in (**J**). Error bars represent mean ± SEM. *n* = 9 per group; *p < 0.05, LFD vs Gabra5^cRNA^ and HFD vs Gabra5^cRNA^ in the intake of LFD; ^#^*p* < 0.05 LFD vs Gabra5^cRNA^ and HFD vs Gabra5^cRNA^ in the intake of HFD. Two-way ANOVA and followed by Bonferroni comparisons test. **r** Macronutrient composition of daily food intake by the mice as described in (**p**).

We evaluated the effect of chronic HFD on GABA_A_R α subunits profiles by qPCR, which revealed that *Gabra5* mRNA (encoding GABA_A_R-α5 subunit) within the MC4R^dBNST^ neurons was greatly reduced after 5 wk of HFD (Fig. 4a). We also found that GABA_A_R-α5 is expressed in post-synaptic neurons within the dBNST that were labeled with trans-synaptic WGA:ZsGreen tracer virally transduced by AgRP neurons (Fig. 4b–e). CRISPR/Cas9-mediated genetic deletion of GABA_A_R-α5 signaling within the MC4R^dBNST^ neurons (extended data Figs. 11a, b and 12) promoted anxiety and depression phenotypes (Fig. 4f–i and extended data Fig. 10a–g). Conversely, either chemogenetic suppression of MC4R^dBNST^ neurons or overexpression of GABA_A_R-α5 within MC4R^dBNST^ neurons protected against HFD-mediated anxiety and depression without side effects on locomotion (Fig. 4j–o and extended data Fig. 10h–n). Together, these findings suggest that GABAergic signaling onto MC4R^dBNST^ neurons regulates HFD-induced anxiety and depression by a mechanism involving GABA_A_R-α5 subunits.

To study the contribution of GABA_A_R-α5 to the control of food intake and body weight, mice with overexpression of GABA_A_R-α5 within the MC4R^dBNST^ neurons were exposed to ad libitum HFD for 3 months. Strikingly, we found that mice with gain-of-function manipulation of GABA_A_R-α5 within the MC4R^dBNST^ neurons exhibited a late onset, but potent, attenuation of food intake and reduction of body weight (Fig. 4p). Furthermore, results from food-preference assays performed during days 86–92 demonstrated a switch of food choice from the HFD towards an LFD (Fig. 4q–r), illustrating that manipulation of the GABA_A_R-α5 signaling within the MC4R^dBNST^ neurons has therapeutic potential.

### The 5-HT_3_R and GABA_A_R-α5 pathways elicit synergistic effects on anxiety-depressive behavior and obesity

**Fig. 5:**
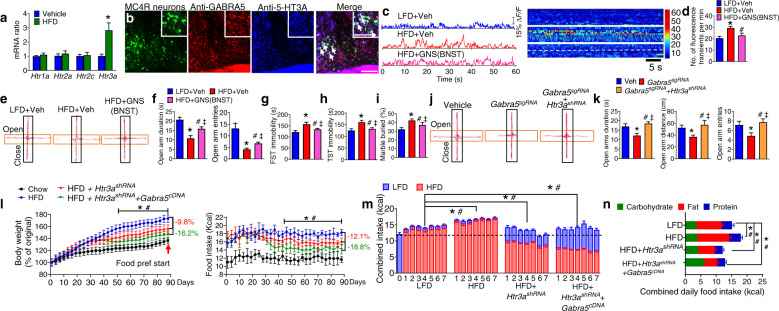
Synergistic effect of the 5-HT_3_R and GABA_A_R-α5 signaling within the MC4R^dBNST^ neurons on HFD-induced mental deficiency and obesity. **a** Expression level of *Htr1a-3a* and *Htr2c* in the dBNST of WT mice treated with LFD or HFD. **B** Co-localization of MC4R neurons with both GABA_A_R-α5 and 5-HT_3_A signaling within the dBNST. Inset in (**b**) is a magnified view showing colocalized neurons with arrow heads. Scale bar in (**b**), 150 μm. scale bar in inset of (**b**), 50 μm. Error bars represent mean ± SEM. *n* = 9 per group; *p < 0.05; two-way ANOVA and followed by Bonferroni comparisons test. **c** Examples of in vivo fluorescence traces in GNS or vehicle-treated *Mc4r*^*Cre*^ mice with *AAV9-FLEX-GCaMP6f* into the dBNST followed with a chronic treatment of HFD or LFD (left). Right, Heat map of color-coded changes of fluorescence intensity in the mice as described in (**C**). **d** The number of fluorescence transients detected in MC4R^dBNST^ neurons. Error bars represent mean ± SEM. *n* = 10 per group; *p < 0.05, LFD + Veh vs HFD + Veh; ^#^*p* < 0.05, HFD + Veh vs HFD + GNS; one-way ANOVA and followed by Tukey comparisons test. **e**–**i** The EPM (**e**, **f**), FST (**g**), TST (**h**), and MBT (**i**) test by the mice as described in (**c**). Error bars represent mean ± SEM. *n* = 7–9 per group; *p < 0.05, LFD + Vehicle vs HFD + Vehicle; ^#^*p* < 0.05, HFD + Vehicle vs HFD + GNS; ^‡^*p* > 0.05, LFD + Vehicle vs HFD + GNS. one-way ANOVA and followed by Tukey comparisons test. **j**, **k** OFT test by *Mc4r*^*Cre*^*::Rosa26*^*Cas9*^ mice with a bilateral injection of either vehicle (Vehicle), *AAV9- FLEX-Gabra5*^*sgRNA*^*-tdTomato* (Gabra5^sgRNA^), or *AAV9-FLEX-Gabra5*^*sgRNA*^*-tdTomato* and *AAV9-FLEX-Htr3a*^*shRNA*^*-EYFP* (Gabra5^sgRNA^ + Htr3a^shRNA^) into the dBNST. Error bars represent mean ±  SEM. *n* = 6–8 per group; *p < 0.05, Vehicle vs Gabra5^sgRNA^; ^#^*p* < 0.05, Gabra5^sgRNA^ vs Gabra5^sgRNA^ + Htr3a^shRNA^; ^‡^*p* > 0.05, Vehicle vs Gabra5^sgRNA^ + Htr3a^shRNA^; one-way ANOVA and followed by Tukey comparisons test. **L** Body weight (left) and daily food intake (right) in LFD or HFD-treated *Mc4r*^*Cre*^*::Rosa26*^*Cas9*^ mice with injection of *AAV9-FLEX-Htr3a*^*shRNA*^*-EYFP* or combined *AAV9-FLEX-Gabra5*^*sgRNA*^*-tdTomato* and *AAV9-FLEX-Htr3a*^*shRNA*^*-EYFP* (Gabra5^sgRNA^ + Htr3a^shRNA^) into the dBNST. Error bars represent mean ±  SEM. *n* = 9 per group; *p < 0.05, HFD vs HFD + Htr3a^shRNA^; two-way ANOVA and followed by Bonferroni comparisons test. **m** Combined intake of HFD and LFD in a food preference test following the assay in the mice described in (**l**). Error bars represent mean ±  SEM. *n* = 9 per group; *p < 0.05, LFD vs Htr3a^shRNA^ and HFD vs Htr3a^shRNA^ in the intake of LFD; ^#^*p* < 0.05 LFD vs Htr3a^shRNA^ and HFD vs Htr3a^shRNA^ in the intake of HFD. Two-way ANOVA and followed by Bonferroni comparisons test. **n** Macronutrient composition of daily food intake by the mice described in (**l**).

We also examined the HFD-mediated expression profile of 5-HT receptor subtypes within the MC4R^dBNST^ neurons and found that *Htr3a* (encoding the 5-HT_3_R subunit A) showed the most robust elevation upon long-term treatment of HFD (Fig. 5a). Our immunostaining results indicated that both GABA_A_R-α5 and 5-HT_3_R receptors were co-localized within MC4R^dBNST^ neurons (Fig. 5b). In contrast, MC4R^dBNST^ neurons did not co-express 5-HT1A (extended data Fig. 13a–f). The in vivo fiber photometry results revealed that MC4R^dBNST^ neurons are hyperexcitable after chronic treatment with the HFD, an effect that was blocked by treatment of granisetron (GNS), a selective 5-HT_3_R antagonist (Fig. 5c, d). Infusion of GNS into the dBNST effectively rescued HFD-induced anxiety and depression (Fig. 5e–i and extended data Fig. 13g–k).

We further examined the functional relevancy of GABA_A_R-α5 and 5-HT_3_R in the regulation of mental states and body weight using combined CRISPR/Cas and RNAi techniques. *AAV9-FLEX-Gabra5*^*sgRNA*^*-tdTomato* injected into the dBNST of *Mc4r*^*Cre*^*::Rosa26*^*Cas9-EGFP*^ mice (extended data Fig. 14a–b) led to elevated anxiety and depression, which was fully prevented by additional injection of *AAV9-FLEX-Htr3a*^*shRNA*^-EYFP into the dBNST (extended data Fig. 13l–o). However, neither of these manipulations altered basal locomotion (extended data Fig. 13s, t). To assess whether the manipulation of *Htr3a* within MC4R^dBNST^ neurons contributes to the regulation of HFD-induced anxiety-depressive behavior and body weight, mice deficient in *Htr3a* within MC4R^dBNST^ neurons were subjected to chronic exposure to ad lib HFD for 3 months. We found that loss of 5-HT_3_R from MC4R^dBNST^ neurons fully rescued anxiety and depression after 35 days of viral treatment (Fig. 5j, k and extended data Fig. 13p–w). More strikingly, these mice exhibited a late onset, but potent, attenuation of food intake and reduction of body weight after treatment of HFD (Fig. 5l). Furthermore, results from food-preference assays demonstrated a switch of food choice from HFD towards an LFD (Fig. 5m, n). Together, these results indicate that genetic suppression of 5-HT_3_R or enhancement of GABA_A_R-α5 within MC4R^dBNST^ neurons is sufficient to revert mental dysfunction which in turn normalizes food consumption and body weight.

### A combo pharmacotherapy alleviates HFD-induced anxiety/depression and reverses obesity

**Fig. 6:**
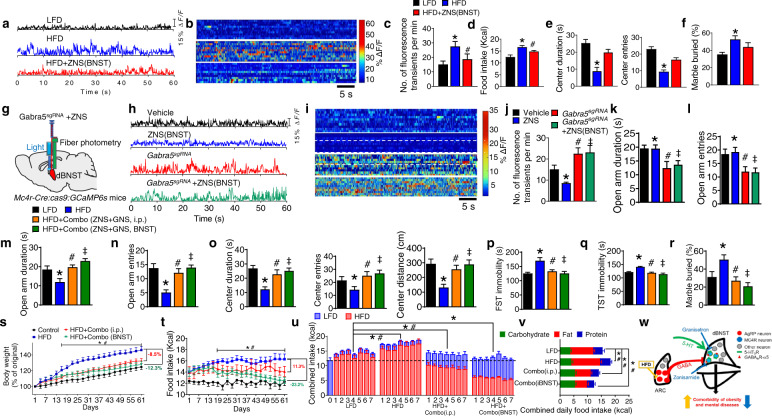
Combination of zonisamide and granisetron alleviates HFD-induced mental dysfunctions and rescues obesity. **a** Examples of in vivo fluorescence traces in ad-lib fed *Mcr4*^*Cre*^ mice with *AAV9-FLEX-GCaMP6f* into the dBNST followed with a chronic treatment of LFD or HFD or HFD combined with injection of ZNS into the dBNST. **b** Heat map of color-coded changes of fluorescence intensity in the mice as described in (**a**). **c** The numbers of fluorescence transients detected in MC4R^dBNST^ neurons of the mice as described in (**a**). **d** Daily food intake by WT mice with the chronic treatment of LFD or HFD or HFD combined with injection of ZNS into the dBNST. Error bars represent mean ± SEM. *n* = 7–9 per group; *p < 0.05, LFD vs HFD; ^#^*p* > 0.05, HFD + ZNS vs LFD. One-way ANOVA and followed by Tukey comparisons test. **d**, **e** OFT (**d**) and MBT (**e**) assays by the mice as described in (**a**). **f** Diagram showing in vivo fiber photometry analysis of Mc4r^dBNST^ neurons in response to *Gabra5*-KO and ZNS. **g** Examples of in vivo fluorescence traces in ad-lib fed *Mc4r*^*Cre*^*::Rosa26*^*Cas9*^ mice with bilateral injection of either vehicle, *AAV9-FLEX-Gabra5*^*sgRNA*^*-tdTomato* (Gabra5^sgRNA^) into the dBNST, followed by microinjection of vehicle or ZNS into the dBNST. **h** Heat map of color-coded changes of fluorescence intensity in the mice as described in (**g**). **i** The numbers of fluorescence transients detected from the MC4R^dBNST^ neurons in mice as described in (**g**). **j**, **k** EPM test of the mice described in (**g**). Error bars represent mean ± SEM. *n* = 7–9 per group; *p < 0.05, Vehicle vs Gabra5^sgRNA^; ^#^*p* < 0.05, Vehicle vs ZNS; ^‡^*p* > 0.05, Gabra5^sgRNA^ vs Gabra5^sgRNA^ + ZNS. One-way ANOVA and followed by Tukey comparisons test. **l**–**q** EPM (**l**, **m**), OFT(**n**), TST(**o**), FST(**p**), and MBT (**q**) assays in WT mice with i.p. or i.c.v injection of both ZNS and GNS into dBNST by chronic HFD feeding. **r**, **s** Body weight (**r**) and food intake (**s**) in mice with the chronic treatment of LFD or HFD or HFD combined with i.p. injection of ZNS and GNS into the dBNST or HFD combined with microinjection of ZNS and GNS into the dBNST during a 7-week treatment of HFD. Error bars represent mean ± SEM. *n* = 7 per group; *p < 0.05, HFD vs HFD + ZNS + GNS (i.p.); ^#^*p* < 0.05, HFD vs HFD + ZNS + GNS (BNST); two-way ANOVA and followed by Bonferroni comparisons test. **t** Combined intake of LFD and HFD in mice as described in (**r**) and (**s**) during the last 7 days of the 7-week treatment of HFD. Error bars represent mean ± SEM. *n* = 7 per group; *p < 0.05, LFD vs HFD + ZNS + GNS (i.p.), HFD vs HFD + ZNS + GNS (BNST) and HFD + ZNS + GNS (i.p.) vs HFD + ZNS + GNS (BNST) in the intake of LFD; ^#^p < 0.05, LFD vs HFD + ZNS + GNS (i.p.), HFD vs HFD + ZNS + GNS (BNST) and HFD + ZNS + GNS (i.p.) vs HFD + ZNS + GNS (BNST) in the intake of HFD. Two-way ANOVA and followed by Bonferroni comparisons test. **u** Macronutrient composition of food intake by the mice as described in (**r**). **v** Schematic diagram showing that a cocktail therapy of zonisamide and granisetron by action through the GABA_A_R-α5 and 5-HT_3_R signaling, respectively, within the MC4R^dBNST^ neurons effectively eliminate obesity and mental disorders comorbidity.

Evidence suggests that zonisamide (ZNS), an anti-convulsant medication, suppresses food intake and renders moderate weight loss in various preclinical and clinical trials [55–59]. However, the mechanisms underlying appetite regulation are unknown at both cellular and circuitry level. Here we found that ZNS, when infused into the dBNST, potently suppressed HFD-induced hyperexcitability of MC4R^dBNST^ neurons (Fig. 6a–c). In addition, there was a significant decrease in daily food intake of ZNS-treated mice after chronic HFD feeding (Fig. 6d). Behavioral tests revealed that intra-dBNST treatment with ZNS completely rescued HFD-induced anxiety and depression without affecting locomotion (Fig. 6e, f and extended data Fig. 15a–h). To determine how ZNS acts within the dBNST neural circuit, we examine the neuronal activity of MC4R^dBNST^ neurons upon CRISPR/Cas9-mediated deletion of the GABA_A_R-α5 signaling. The fiber photometry results showed that intra-dBNST infusion of ZNS significantly reduced the activity of MC4R^dBNST^ neurons, whereas ZNS-mediated suppressive effect was nullified by removal of GABA_A_R-α5 from these neurons (Fig. 6g–j). Furthermore, the behavioral results indicated that ZNS-mediated anxiolytic and anti-depression effects were abolished after deletion of *Gabra5* from MC4R^dBNST^ neurons (Fig. 6k, l and extended data Fig. 15i–s). These data suggest that ZNS mediates anxiety and depression responses by acting through the GABA_A_R-α5 signaling in MC4R^dBNST^ neurons.

These encouraging results suggested that combined treatment with ZNS and GNS might confer additive effects over the control of HFD consumption and obesity. To test this hypothesis, mice were subjected to chronic exposure to HFD for 3 months with intra-dBNST infusion of combined ZNS and GNS. Behavioral results indicated that both systemic administration or intra-dBNST infusion of combined ZNS and GNS produced profound anxiolytic and anti-depression effects (Fig. 6m–r and extended data Fig. 15t–v). Notably, this combo treatment of ZNS and GNS reversed diet-induced obesity by reducing total calorie intake (Fig. 6s–u). Both systemic administration or intra-dBNST infusion of combined ZNS and GNS directed food preference from HFD toward a healthy LFD (Fig. 6v). Furthermore, analysis of macronutrient composition showed that co-administration of ZNS and GNS was associated with a reduction of fat consumption along with a moderate increase of carbohydrate intake, resulting in a much healthier nutrient intake profile (Fig. 6w). Thus, combined administration of ZNS and GNS robustly mitigates HFD-induced anxiety/depression-like behavior and further reverses obesity by rebalancing the composition of macronutrient intake.

## Discussion

Until we have a thorough understanding of the underlying neurobiological mechanisms, effective management of obesity and related emotional comorbidity is a challenging task [60–65]. In the present study, we found that chronic HFD treatment leads to desensitization of AgRP neurons in response to hunger, downregulation of GABA_A_R-α5 signaling, and upregulation of 5-HT_3_R within the post-synaptic MC4R^dBNST^ neurons, rendering abnormal hyperactivity of MC4R^dBNST^ neurons with an overall effect on promoting HFD-mediated anxiety and depression (Fig. 6w). Disruption of GABAergic signaling from AgRP neurons led to mental dysfunction, which could be rescued by enhancing GABA_A_R signaling within downstream dBNST neurons. Furthermore, stimulation of post-synaptic MC4R^dBNST^ neurons or genetic inactivation of GABA_A_R-α5 signaling promoted anxiety and depression-like responses. Conversely, genetic enhancement of GABA_A_R-α5 or disruption of 5HT_3_R significantly attenuated mental dysfunctions, which in turn contributed to the mitigation of HFD-associated obesity. Furthermore, pharmacological treatment of two clinically approved drugs, ZNS and GNS, acting through GABA_A_R-α5 and 5HT_3_R, respectively, displayed a strong synergistic effect in reversal of HFD-induced mental phenotype and a robust loss of the body weight by switching macronutrient composition to mixture of HFD with a significant amount of healthy LFD.

We demonstrate effective rescuing effects of both anxiety-depressive deficiency and obesity by facilitating GABA_A_R signaling within the dBNST, genetic suppression of MC4R^dBNST^ neurons, or even a combo pharmacological therapy by administering ZNS and GNS peripherally or directly into the dBNST. The uniqueness of this neural pathway with defined cellular identity and molecular signaling provides mechanistic insights into how the BNST regulates feeding and mental functions [32, 33]. The profound modulation of AgRP^ARC^ → MC4R^dBNST^ circuit and associated physiological and behavioral outcomes by chronic treatment of HFD. A recent study showed that obesity remodels the activity and transcriptional state of a lateral hypothalamic glutamatergic neurons that encode satiety state [66]. Another study indicated that the lateral hypothalamic hypocretin/orexin (Hcrt) neurons were functionally desensitized in obese mice [67]. Take these together, we suggest that HFD-induced obesity was coupled with profound and diversified changes in several neuronal groups. Future efforts of identifying novel circuitry components in the context of the critical AgRP^ARC^ → MC4R^BNST^ circuit and associated receptor signaling cascades would unveil a more complete neural mechanism for obesity and psychotic comorbidity.

Serotonin system is a major contributor to mood regulation, upon which a wide variety of medications for mental disorders were developed [68]. Recent studies showed that several 5-HT receptor subtypes, including 5-HT_1A_, 5-HT_2A_, 5-HT_2C_, play substantial roles in the mitigation of anxiety and depression [34, 68, 69]. Nevertheless, we found that chronic HFD treatment prompted a robust upregulation of 5-HT_3_R within the BNST MC4R neurons. Treatment of a 5-HT_3_R antagonist alleviates HFD-induced anxiety-depressive dysfunction. Furthermore, genetic suppression of 5-HT_3_R signaling attenuates both anxiety and depression phenotypes in HFD-fed mice with a subsequent reduction of food intake and weight loss. These results suggest that 5-HT_3_R within the MC4R^dBNST^ neurons is a unique target for obesity and its mental comorbidity [41].

Our time-course study indicated that prior to development into obesity, animals exposed to chronic HFD treatment display progressive manifestations of anxiety and depression phenotypes (Fig. 1a–f and extended data Fig. 1a–i). On the other hand, the rescuing studies showed that both genetic and pharmacological interventions lead to a significant weight loss after HFD feeding. Taken with previously reported clinical studies [4], we suggest a causal relationship between anxiety-depressive responses and obesity. Collectively, targeting GABA_A_R-α5 and 5HT_3_R within MC4R^dBNST^ neurons facilitates the rescue of HFD-induced anxiety and depression disturbance, which in turn lowers body weight by simultaneously reducing the craving of HFD and enhancing feeding of healthy low-fat diets in mice.

Pharmacological studies suggest that central GABA_A_ receptor signaling exerts prominent influences on feeding in various brain regions [17, 18, 26]. A class of benzodiazepine agonists, approved for anxiety disorder treatment, which acts through a specific binding site on GABA_A_ receptors, induces hyperphagia by enhancing food and taste palatability [26, 70]. In line with this literature, we found that genetic manipulation of GABA_A_R-α5 produced profound anti-obesity effects and attenuation in HFD-induced anxiety and depression in mice. It is noteworthy that HFD-mediated desensitization of AgRP neurons is coupled with a significant reduction of *Gabra5* signaling within the post-synaptic MC4R^dBNST^ neurons (Fig. 1g–i, and Fig. 4a), demonstrating a functionally hierarchal structure of this neural circuit. Furthermore, we observed an additive anti-obesity effect with combined genetic manipulations of 5-HT_3_R and GABA_A_R-α5 within the dBNST^MC4R^ neurons, laying a mechanistic foundation for a novel pharmacological intervention by targeting both signaling pathways for obesity and mental comorbidity.

Past clinical trials in an attempt to establish ZNS, a clinically approved anti-convulsant drug, as an anti-obesity medication were largely unsuccessful, largely due to the elusive pharmacological mechanism and marginal effects on weight loss [55, 56, 58, 59, 71]. We demonstrated here that ZNS acts through GABA_A_R-α5 signaling with a robust effect on weight loss and anxiety-depressive recovery. Combined treatment of GNS and ZNS achieved a strong synergistic effect on the reversal of obesity and coinciding mental dysregulation. Our results provide convincing support for the future clinical study by a cocktail therapy with combined treatment of ZNS and GNS, as well as a more comprehensive drug repurposing pipeline for metabo-psychiatric diseases [72–74].

## Methods

### Animals

All animal care and experimental procedures were approved by the Institutional Animal Care and Use Committees at Baylor College of Medicine. Mice used for data collection were both males and females, at least eight weeks of age. All animals were kept in temperature- and humidity-controlled rooms, in a 12/12-h light/dark cycle, with lights on from 7:00 AM–7:00 PM. Food and water were provided ad libitum. A 40% Kcal fat diet was used (TD.95217, Envigo, Indianapolis, IN) for in vivo fiber photometry and behavioral study. Another 60% Kcal fat diet was used (TD.06414, Envigo, Indianapolis, IN) for chronic food intake, body weight, and food preference study.

Genetic Mouse Model. *Agrp*^*DTR/+*^ mice [28], *Agrp*^*Cre*^ mice [30], *Agrp*^*nsCre*^ mice [29], Gad1l^ox/lox^*::Gad2*^*lox/lox*^ mice [29], *Npy*^*GFP/+*^ mice [75], Ai14 (or *Rosa26*^*tdTomato*^) mice [76], Ai32 mice [76], *Mc4r*^*Cre*^ mice [15], and *Rosa26*^*Cas9*^ mice [77] were produced as described previously. All mice are on a C57Bl/6 background with at least eight generations backcrossed.

### Brain surgery and viral injections

Animals were anesthetized by isoflurane and placed on a stereotaxic frame. A 26-gauge stainless steel guide cannula (PlasticsOne, USA) with an infusion dummy cannula was placed into the dBNST (coordinates: +1.0 mm, 1.0 mm lateral from bregma and depth −4.3 mm). To target the ARC the coordinate is: (−1.7 mm, 0.25 mm. 5.9 mm). To target the third ventricle the coordinate is: (−1.7, 0. 5.6 mm). The following virus was used in the different experiments: *AAV9-FLEX-GCaMP6f, AAV9-DIO-WGA-ZsGreen, AAV9-DIO-GTB*, *EnvA G-deleted RV-mCherry, AAV9-FLEX-hM3Dq-mCherry, AAV9-FLEX-hM4Di-mCherry, AAV9-Gabra5*^*sgRNA*^*-tdTomato, AAV9-FLEX-Gabra5*^*shRNA*^-*tdTomato, AAV9-FLEX-Gabra5*^*cRNA*^*-tdTomato, AAV9-FLEX-Htr3a*^*shRNA*^*-EYFP*, injected bilaterally (0.3 μl per each side) through Hamilton syringe (31 gauge, Model 75 Small RN Syringe) using a pump (UMP3 UltraMicroPump, Sarasota, FL, USA).

### Pharmacology

To disrupt GABAergic inputs from AgRP neurons in adult mice, microinjection of NB124 [29] (two injections of 0.4 mg/side, 2 days apart; Calbiochem) in 6-week-old *Agrp*^*DTR/DTR*^ mice was performed. To chronically infuse bretazenil (BTZ) to the dBNST Alzet micro-osmotic pumps (model 1002, Durect, Cupertino, CA, USA) loaded with 100 μl of BTZ (3 mg/ml in saline plus 10% DMSO; Sigma-Aldrich, St Louis, MO, USA) were implanted subcutaneously on the back of anesthetized mice at 5-month old. These minipumps dispense 0.25 μl/h. Specifically, ad libitum fed mice will be euthanized in the morning (~9 am) 30 days after NB124 injection to harvest the brain tissue for c-Fos staining. The cannulas (28 gauge, Plastics One) were placed into either the third or fourth ventricles under anesthesia, and the subcutaneous Alzet minipumps were connected to the cannulas by tubing (PE60; Stoelting, Wood Dale, IL, USA) that was threaded under the skin to help prevent the mice from dislodging it. To chemogenetic activation or inactivation neurons the mice will be administrated with CNO by bilateral microinjection (0.4 ng/side) or i.p. injection (1 mg/kg). To study the effect of 5-HT receptors on anxiety and depression the 5-HT receptors antagonist GNS (0.1 ug/side, Sigma-Aldrich) were i.c.v. administrated.

### Food intake and body weight

Mice were individually housed and allowed to acclimate for 7 d before experiments were initiated. Consumption of diet pellets and body weight were measured on a daily basis throughout the experimental period.

### Open-field test

To assess locomotor activity and anxiety levels, animals were gently removed from the home cage and placed singly into an open-field arena (Plexiglas cage, 50L × 50 W × 40H cm). An automated video-tracking system (Ethovision by Noldus) was used to track multi subjects simultaneously. After the experiment gently returns each mouse to the respective home cage. To avoid olfactory cues the arena (usually 50% EtOH) was carefully wiped after every running. The central area delineated virtually with Ethovision software, is taken as an imaginary inner square (30 × 30 cm) of the open filed. The amount of time spent and distance traveled in the center of the chamber compared to the edges, as well as the frequency into center zone and total distance traveled across a session of 5 min were calculated.

### Elevated plus maze test

To assess anxiety levels, animals were removed from their home cages and placed individually into an elevated plus maze. The plus maze consisted of four elevated arms (30L × 5W cm) intersected at right angles. Two opposite arms were enclosed by 15–cm high walls, and the other two were open (no walls). The structure was elevated 60 cm above the floor and mice were placed on the 5 × 5 cm intersection of the maze facing into an arm with walls to start a trial. The Ethovision system was used to track multi subjects simultaneously. After the experiment gently returns each mouse to the respective home cage. To avoid olfactory cues the arena (usually 50% EtOH) was carefully wiped after every running. The amount of time the mice spent and distance traveled in the open arms of the maze, as well as the frequency into open arms and total distance traveled throughout a 5-min session were calculated.

### Tail suspension test

To assess depressive-like behaviors, mice were done with a tail suspension test. Once each piece of tape was attached to a mouse tail stick the middle portion of the tape to the horizontal bar. Once all tape is applied, start the recording and identify the session before the mice are suspended. The whole suspension lasted 6 min. At the end of the session the animals were returned to their home cage and carefully remove the tape from each tail by gently pulling it off. The immobility time was assessed using the Ethovision tracking system.

### Forced-swim test

To assess depressive-like behaviors, mice were performed a forced-swim test. The cylindrical tanks (40H × 20D cm) were used in the mouse forced-swim test which are constructed of transparent Plexiglas. A water-resistant infrared thermometer was used to monitor the water temperature. Bring the animals into the testing room for 30 min rest. Then mice were held by the tail and gently placed in the container with ~40 cm of water (23–25 °C) for 6 min. After the experiment removes the animals from the water by their tails and dry them with drying paper and place back into their home cage. The immobility time was assessed using the Ethovision tracking system.

### Marble burying test

To assess depressive-like behaviors, mice were performed a MBT. A rat cage was filled to 5 cm of corn-based animal bedding. Twenty-eight black, glass marbles were evenly spaced within the box, and mice were placed in the center with test sessions of 30 min. At the end of the sessions, the numbers of buried marbles (at least to 2/3 depth) were counted for statistical analysis.

### Optogenetics

For in vivo optogenetic stimulation the optic fiber was assembled as described following the protocol [78]. The mice were unilaterally implanted optic fiber in the dBNST. The 1.25 mm ceramic ferrule with 200 µm fiber compatible with 1.25 mm optogenetics patch cables connected to spectralynx (Neuralynx, Inc, USA) was inserted into the cannula with its projection 0.5 mm below the guide cannula. Blue light was shined into the dBNST at 20 Hz frequencies during the behavioral tests.

### In Vivo fiber photometry

For in vivo fiber photometry, *Agrp*^*Cre*^ *or Mc4r*^*Cre*^ mice were injected *AAV9-FLEX-GCAMP6f* into the ARC or dBNST. Animals were allowed to recover for at least 2 weeks before experiments proceeded. Optic probes were assembled and implanted following the previous protocol. The 488 nm laser was used to excite GCaMP6 through the multi-mode fiber patch cord and the QE Pro detector (Ocean optics) was used to collect the photons emitted from the tissue through the multi-mode detection fiber patch cord. The OceanView software (Ocean optics) was used to acquire the data. Spectral channel (500–543 nm) was selected for GCaMP6. The integrated photon count was used as a measure of intensity. The spectrum data were recorded continuously at 10 Hz sampling frequency. The percentage ∆*F*/*F* was calculated by 100 × (*F*− *F*mean)/*F*mean, where *F*mean was the mean fluorescence intensity throughout the entire acquisition fragment. The detection threshold for a fluorescence transient was defined as *µ* + 3*σ*, where *µ* and *σ* were the mean and the standard deviation of the fluorescence baseline period. The fluorescence transients during baseline were randomly sampled (40–45 s). For the heat map the recorded data were saved as ASCII files and opened in MATLAB. And the heat map can be plotted by Matlab. All Data were analyzed offline using Excel, Matlab, and Prism.

### In vitro electrophysiological recordings

The mice were handled and kept in artificial cerebrospinal fluid (aCSF) as described recently [79]. Animals were subjected to anesthesia, and the handling protocol of the local committee was followed. The slices containing the dBNST in the coronal plane (250–300 μm) were prepared. In most case, we have used the same mouse first for the in vivo experiments and immediately after for combined electrophysiology and optogenetics in vitro in order to minimize the number of mice. The whole brain was quickly dissected into ice-cold oxygenated aCSF containing (in mM): 130 NaCl, 5 KCl, 2.4 CaCl_2_, 1.3 MgSO_4_, 1.25 KH_2_PO_4_, 10 glucose, and 20 NaHCO_3_, pH 7.4 with NaOH, bubbled with a mixture of 95% O_2_ and 5% CO_2_. Then the brain was cut coronally into 300-μm slices on a microtome (VTA-1000S; Leica). Slices containing the aDRN were transferred to an incubation chamber filled with aCSF and incubated for at least 1 h at room temperature (24–26 °C). At RT, the slices were transferred to a recording chamber on the stage of a fluorescence microscope (BX51WI, Olympus) and maintained immersed and continuously superfused with aCSF at 4–5 ml/min. Patch electrodes were pulled on a micropipette puller (P-97, Sutter Instruments) from borosilicate capillaries (GC150-10, Harvard Apparatus). The pipettes (5–12 MΩ) contained (in mM): 97.5 K-gluconate, 32.5 KCl, 1 MgCl_2_.6H_2_O, 40 HEPES, 0.5 Na-GTP, 2 Mg-ATP, and 0.5 EGTA, pH 7.4. Recordings were made with a MutiClamp 700B amplifier (Molecular Devices). The dBNST neurons were subjected to electrophysiological recordings. The action potentials (APs) were recorded in response to either depolarizing current injection or light expose or DT treatment. Data were acquired and analyzed using Spike2 7.04 software (Cambridge Electronic Design). Patchmaster, Clampfit, Mini Analysis, Origin and Microsoft Excel software were used for data acquisition and subsequent analyses. The current signals were filtered at 1 kHz and the sampling interval was 50−200 μs. The number of experiments (*n*) and means ± SEM are indicated. Comparisons were assumed significant if *p* < 0.05.

### FACS

The tdTomato-positive or EYFP-positive neurons were isolated by a standard flow cytometry method as described [29]. Briefly, brain tissues containing the ARC or the dBNST were collected into 0.5 mL of ice-cold HBSS buffer, then transferred to 37 °C warmed dispase buffer (BD Biosciences), and incubated for 15 min. During the incubation period, tissues were gently pipetted up and down until completely dissociated. Next, 0.5 mL ice-cold FACS buffer [10% (wt/vol) glucose, 0.5 mM EDTA, and 3 mg/mL BSA] was added to stop the dispase-mediated dissociation process. Cells were pelleted by centrifuging for 2 min at 3000 × *g* at 4 °C. The pellets were resuspended in 0.5 mL of ice-cold FACS buffer and then filtered into polypropylene tubes (Falcon) before the FACS procedure.

### Quantitative real-time PCR

Total RNA was isolated using Trizol reagent (Life technologies) and treated with RQ1 DNase (Promega). First strand cDNA was synthetized by superscript II reverse transcriptase (ThermoFisher Scientific) according to the manufacturer’s protocol and quantitative real-time PCR was performed using Taqman Primers. All samples were run in triplicate 25 µl reaction (GoTaq® colorless master mix, Promega) using CFX96 q-PCR system (Bio-Rad) and normalized to the GAPDH using the ΔΔCt method.

AgRP Neurons Counting. *Agrp*^*DTR/+*^*::Npy*^*GFP/+*^ mice were killed and perfused transcardially with ice-cold PBS buffer (pH 7.4) containing 4% (wt/vol) paraformaldehyde (Alfa Aesar). Brains were collected and postfixed overnight under 4 °C in a fixation buffer containing 3% paraformaldehyde. About 90 free-floating sections (20 µm) were cut by a microtome (Thermo Fisher) in the arcuate nucleus (ARC) of the hypothalamus and then soaked in ice-cold PBS buffer (pH 7.4). After mounting the sections, fluorescent images were captured by a digital camera mounted on a DMI6000B microscope (Leica). The whole ARC was divided into nine segments from anterior to posterior ARC. The neuron numbers of each section were manually counted by using ImageJ plugin Cell Counter (NIH).

### Zonisamide (ZNS) treatment

To demonstrate that ZNS can attenuate the hyperactivity of the dBNST Mc4r neurons, three groups of young male *Mc4r*^*Cre*^ mice were injected with *AAV9-FLEX-GCAMP6f* into the dBNST. After recovery, cannula was implanted above the dBNST. The mice were randomly assigned into three groups (LFD, HFD, HFD + ZNS, *n* = 8 for each group) and fed with LFD or HFD. For HFD + ZNS group, ZNS (5 µg/side/mouse/day) was i.c.v. injected into the dBNST for 7 days, while the other two groups were injected with the same volume of vehicle. In vivo fiber photometry was conducted at day 0, 3, 7.

To test whether modulation of 5-HT signaling in the dBNST can sensitize ZNS’s effects on anxiety and depression, four groups of young mice (LFD, HFD, HFD + ZNS, HFD + ZNS + GNS, *n* = 11 for each group) were implanted with cannula above the dBNST for drug delivery and fed with LFD or HFD. For HFD + ZNS group, ZNS was i.c.v injected into the dBNST (5 ug/side/day/mouse), and ZNS (5 µg/side/day/mouse) and GNS (0.5 µg/side/day/mouse) for HFD + ZNS + GNS group, while the LFD and HFD groups were microinjected with the same volume of vehicle.

To elucidate the effect of ZNS is dependent on *Gabra5* signaling, young *Mc4r*^*Cre*^ mice were injected with *AAV9-FLEX-GCAMP6f* and *Gabra5*^*sgRNA*^ into the dBNST, fed with HDF, and separately housed. After recovery, cannula was implanted above the dBNST. After 21 days of microinjection of ZNS at 5 µg/mouse/day, the anxiety and depression behavior paradigm were tested in 10 days. In vivo fiber photometry was conducted at day 0, 7,14, 21.

For long-term drug combination study, four groups of young mice were bilaterally implanted with cannula above the dBNST and separately housed. After recovery, mice were fed with control diet (*n* = 11) or HFD (*n* = 35) for 90 days. For HFD + ZNS group (*n* = 12), 5 µg/side/day/mouse were i.c.v. injected into the dBNST, and 5  µg/side/day/mouse+0.5 ug/side/day/mouse for HFD + ZNS + GNS group (*n* = 12), while the control and HFD groups were microinjected with the same volume of vehicle. Body weight and food intake were measured daily. For food preference study, after the 90-day trial, the mice were presented with both LFD and HFD. The daily food intake of the two diets for each single-caged mouse was recorded.

### Immunohistochemistry

Immunostaining was performed as described with modification [29]. Mice were killed and perfused transcardially with ice-cold PBS buffer (pH 7.4) containing 3% (wt/vol) paraformaldehyde (Alfa Aesar) and 1% glutaraldehyde (Sigma). Brains were collected and postfixed overnight under 4 °C in a fixation buffer containing 3% paraformaldehyde. Free-floating sections (20 μm) were cut by a microtome (Thermo Fisher) and then blocked with 5% (wt/vol) normal donkey serum in 0.1% Triton X-100 (TBST buffer, pH 7.2) for overnight. For each different assay, either rabbit anti-AgRP (1:500 dilution; Phoenix Pharmaceuticals), rabbit anti-Fos (1:1,500 dilution; EMD Millipore), mouse anti-CRF (1:1000 dilution; EMD Millipore), goat anti-5HT3A (1:300 dilution; Abcam), goat anti-5HT1A (1:300 dilution; Abcam), rabbit anti-GABAR-A5 (1:400 dilution; Abcam), chicken anti-GFP (1:500 dilution; Life Technologies) was applied to the sections for overnight incubation under 4 °C, followed by 4 × 15-min rinses in the TBST buffer. Finally, sections were incubated with Alex Fluor 488-conjugated secondary antibody (1:1000 dilution; Jackson Immunolab) or Alex Fluor cy3-conjugated secondary antibody (1:1000 dilution; Jackson Immunolab) for 2 h at room temperature, followed by 4 × 15-min rinses in TBST buffer. For mounted sections, fluorescent images were captured by a digital camera mounted on a DMI6000B microscope (Leica). All images were analyzed by the ImageJ software.

### Liquid chromatography with tandem mass spectrometry

To evaluate the potential diffusion of dBNST-administered BTZ into surrounding brain regions, LC-MS was applied to calculate the BTZ concentrations after injection into the dBNST for 24 h. The LC-MS was performed by the Metabolomics Core at Baylor College of Medicine. Four punches containing the dBNST, vBNST, LSv, and IPACM from fresh brain tissues were collected with a thickness of 1.0 mm by brain matrix. The wet weights of the brain tissue samples ranged from 8.1 to 15.5 mg. For tissue homogenization, the samples were mixed with cold (−20 °C) methanol:water (1:1, v/v) containing 60 ppm of the internal standard 4-nitrobenzoic acid in a ratio of 1 mg tissue:10 µL of solvent per mg of tissue [ratio 1:10 (w/v)]. The disruption of the tissue was achieved using a Benchmark Bead Blaster Refrigerated Microtube Homogenizer, 115 V (Benchmark, US) for 2 min. 100 µL of the resulting homogenate was transferred into a 1.5 mL Eppendorf and mixed with 320 µL of cold (−20 °C) methanol. The samples were then vortex-mixed for 2 min, followed by air drying. Subsequently, dried samples were reconstituted in 200 µL of 50:50 methanol: water (0.2% formic acid). After centrifugation at 4000 × *g* at 20 °C for 20 min, 100 µL of the sample was divided into two UHPLC-MS vials with insert (10 µL/each) to inject them directly into the system for LC-MS analyses in positive and negative ionization modes.

BTZ concentrations from the brain tissue samples were acquired using liquid chromatography-mass spectrometry (LC-MS/MS; 6495 QQQ Triple Quadrupole MS, Agilent Technologies). The analysis was carried out on high-performance liquid chromatography (HPLC) system equipped with a quaternary pump, a degasser, an auto sampler, and a column compartment. Mass spectrometric detection was carried out using triple coupled quadrupole equipped with an electron spray ionization (ESI) source. The data acquisition was under the control of Mass Hunter software. The separation of BTZ was achieved using an RRHD SB-CN column (1.8 μm, 3.0 × 100 mm, Agilent Technologies). The mobile phase consists of 0.1% formic acid (A) in water and acetonitrile (B) with gradient elution at a flow rate of 0.3 ml/min. Gradient is spanning 2% B to 98% B over 15 min followed by 98% B to 2% B for 1 min. The typical operating source condition for MS scan in positive ESI mode was optimized (*m/z* 417.28, protonated BTZ).

To evaluate the effects of DT diffusion in the dBNST and surrounding brain regions, the relative score for DT concentration by LC-MS was calculated 24 h after DT injection into the dBNST. The LC-MS was performed by the Clinical and Translational Proteomics Service Center at the University of Texas Health Science Center. The brain tissues were punched 24 h after DT (0.4 ng) injection into the dlDRN. Four punches containing the dBNST, vBNST, LSv, and IPACM were collected with a thickness of 1.0 mm by brain matrix. The brain tissue lysates were subjected to acetone precipitation; proteins were precipitated at –20 °C for three hours. After centrifugation (12,000 × *g* × 5 min), the pellets were resuspended in 10 ml of 150 mM Tris-HCl, pH 8.0, denatured and reduced with 20 ml of 9 M urea, 30 mM DTT in 150 mM Tris HCl, pH 8.0, at 37 °C for 40 min, then alkylated with 40 mM iodacetamide in the dark for 30 min. The reaction mixture was diluted 10-fold using 50 mM Tris-HCl pH 8.0 prior to overnight digestion at 37 °C with trypsin (1:20 enzyme to substrate ratio). Digestions were terminated by adding an equal volume of 2% formic acid, and then desalted using Waters Oasis HLB 1 ml reverse-phase cartridges according to the vendor’s procedure. Eluates were dried via vacuum centrifugation.

About 1 microgram of the tryptic digest (in 2% acetonitrile/0.1% formic acid in water) was analyzed by LC/MS/MS on an Orbitrap Fusion Tribrid mass spectrometer (Thermo Scientific) interfaced with a Dionex UltiMate 3000 Binary RSLCnano System. Peptides were separated onto a Acclaim PepMap C18 column(75 mm ID × 15 cm, 2 mm) at a flow rate of 300 nl/min. Gradient conditions were: 3–22% B for 120 min; 22–35% B for 10 min; 35–90% B for 10 min; 90% B held for 10 min,(solvent A, 0.1% formic acid in water; solvent B, 0.1% formic acid in acetonitrile). The peptides were analyzed using data-dependent acquisition method, Orbitrap Fusion was operated with measurement of FTMS1 at resolutions 120,000 FWHM, scan range 350–1500 *m/z*, AGC target 2E5, and maximum injection time of 50 ms; During a maximum 3 s cycle time, the ITMS2 spectra were collected at rapid scan rate mode, with CID NCE 35, 1.6 *m/z* isolation window, AGC target 1E4, maximum injection time of 35 ms, and dynamic exclusion was employed for 60 s.

The raw data files were processed using Thermo Scientific Proteome Discoverer software version 1.4, spectra were searched against the Uniprot Mus musculus plus DT database using Sequest HT search engine. Search results were trimmed to a 1% FDR using Percolator. For the trypsin, up to two missed cleavages were allowed. MS tolerance was set 10 ppm; MS/MS tolerance 0.6 Da. Carbamidomethylation on cysteine residues was used as fixed modification; oxidation of methione as well as phosphorylation of serine, threonine and tyrosine was set as variable modifications.

### Western blots

For western blots, frozen punched dBNST brain tissues (*n*  = 6 from WT, and *n* = 4 for cas9 mediated knockoff of *Gabra5*, ~20 mg) were thawed and homogenized in 100 µl RIPA lysis buffer supplemented with protease inhibitor cocktail (Millipore Inc., Billerica, MA, USA). The lysates were centrifuged at 14,000 × *g* at 4 °C for 30 min and the supernatants were collected and stored at −20 °C until use. Protein concentration was determined by the Bio-Rad DC protein assay (Bio-Rad Laboratories Inc., Hercules, CA, USA). Each sample of 50 µg protein was separated by 10% SDS-PAGE electrophoresis using 100 Volt for 2 h and then transferred to a PVDF membrane at 100 mA for 2 h. The membrane was blocked with 5% non-fat dry milk for 1 h at room temperature and then incubated overnight at 4 °C with primary antibodies diluted as followed:

GABRA5 (1∶1000) (AB9678, Millipore, Billerica, MA, USA), beta-actin (1∶10,000) (MAB1501, Millipore, Billerica, MA, USA). On the next day, after washing three times with 0.05% Tween-20 and phosphate-buffered saline, the membrane was incubated with the corresponding horseradish peroxidase (HRP) conjugated secondary antibody for 1 h at room temperature. Blots were then developed using an ECL Plus Kit (Millipore) on Fuji Medical X-ray film and scanned using a Bio-Rad 6500 scanner. Optical density was quantified with Quantity One software (Bio-Rad).

### Statistical analyses

Data were analyzed by one-way ANOVA or two-way ANOVA with the post hoc as appropriate. Statistical analyses were performed using Prism software (GraphPad Software) Results were considered significantly different at *P* < 0.05. All data are presented as mean ± SEM.

## Supplementary information

Extended Data
