# Supplementary material for: Reciprocal control of obesity and anxiety–depressive disorder via a GABA and serotonin neural circuit
Source: Mol Psychiatry. 2021 Mar 26;26(7):2837–53. doi: 10.1038/s41380-021-01053-w (PMC8505263; doi:10.1038/s41380-021-01053-w)
Supplement: Supplementary file 1 — Extended Data [file 41380_2021_1053_MOESM1_ESM.pdf]

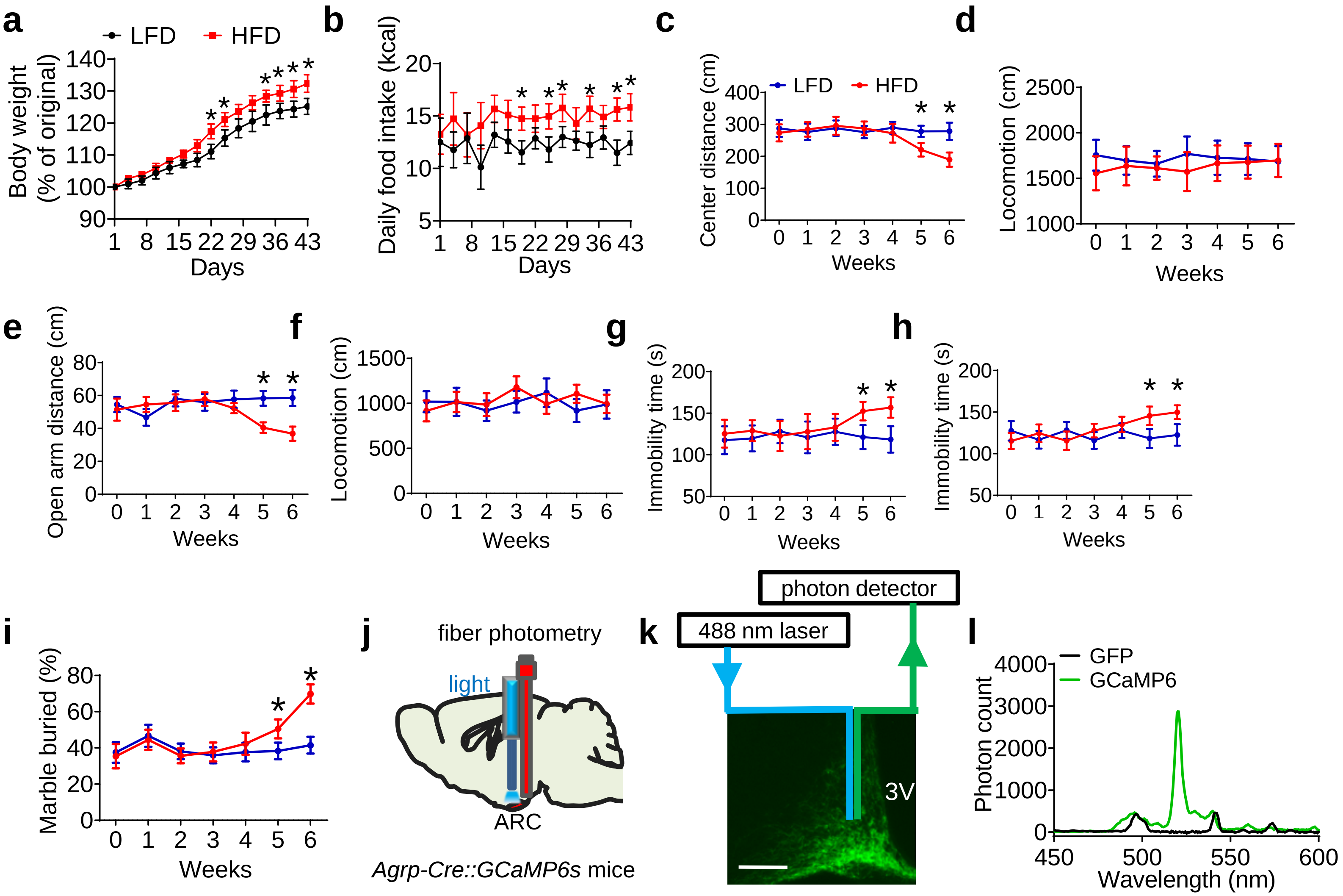

Extended Data Fig. 1. HFD-induced anxiety and depression correlates with the desensitization of AgRP neurons.

a and **b**, Food intake (**a**) and body weight (**b**) of LFD and HFD treated WT mice. **c**-**f**, **c,** Distance travelled in the center zone. **d**, Total distance travelled in the open field in OFT. **e**, Distance travelled in the open arm. **f**, Total distance travelled in EPM. **g**-**i**, FST (**g**), TST (**h**), MBT (**i**) of LFD and HFD treated WT mice, Error bars represent mean ± SEM. n = 8 per group; *p < 0.05, BT vs DT+BTZ; one-way ANOVA and followed by Tukey comparisons test. **j**, Fiber photometry assay revealing the activity of AgRP neurons of *Agrp*^{Cre} mice with injection of *AAV9-FLEX-GCaMP6f* into the ARC (right). **k**, *AAV9-FLEX-GCaMP6f* was injected into the ARC of *Agrp*^{Cre} mice to allow expression of GCaMP6f, a fast-acting calcium sensor, within AgRP^{ARC} neurons. Scale bar, 250 µm. **l**, Fluorescence of GCaMP6f and brain autofluorescence measured from the ARC of *Agrp*^{Cre} mice and WT mice, respectively.

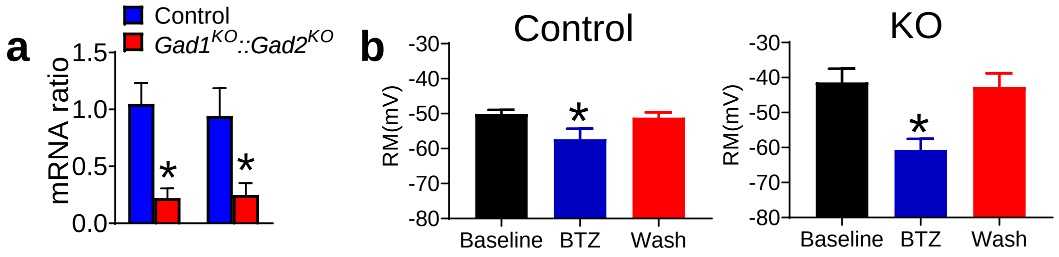

Extended Data Fig 2. AgRP neurons send GABAergic projection to the dBNST.

a, Real-time qPCR analysis of transcript levels of *Gad1* and *Gad2* expressed in AgRP neurons from the control and KO mice. **b**, Resting membrane potential of the post-synaptic dBNST neurons before, during, and after treatment with BTZ (50 µM) from *Agrp*$^{nsCre/+}$::Ai14 mice (Left) or *Agrp*$^{nsCre/+}$::*Gad1*$^{lox/lox}$::*Gad2*$^{lox/lox}$::Ai14 mice (Right) after co-injection of NB124 and *AAV-DIO-WGA:ZsGreen* into the ARC.

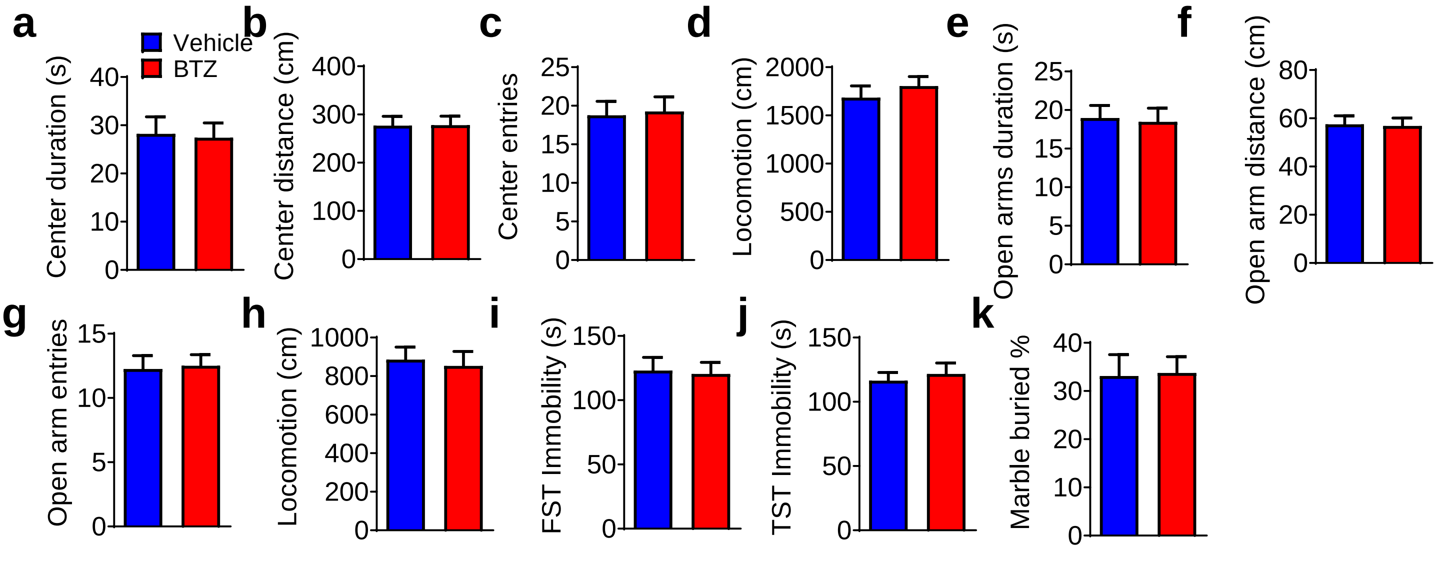

Extended Data Fig. 3. Behavioral responses upon infusion of BTZ into the dBNST.

a Behavior test of in ad-lib fed wild type mice with bilateral microinjection of either vehicle, BTZ into the dBNST. OFT (**a**-**d**), EPM (**e**-**h**), FST (**i**), TST (**j**), and MBT (**k**) assays. Error bars represent mean ± SEM. n = 7-9 per group; p>0.05; two-way ANOVA and followed by Bonferroni comparisons test.

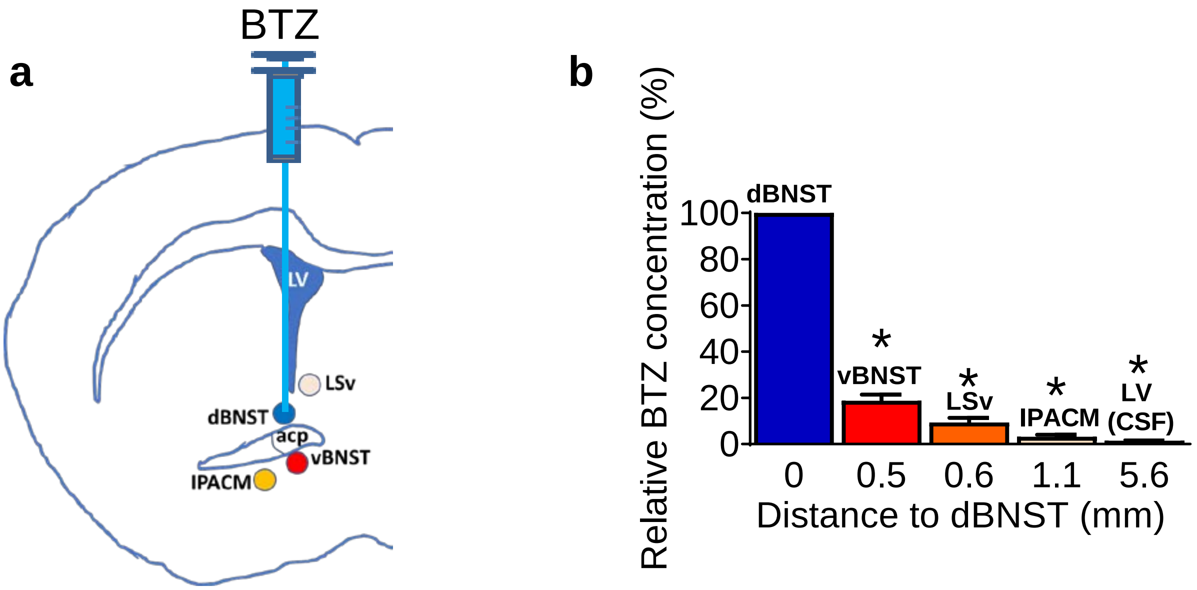

Extended Data Fig. 4. Quantification of the diffusion in brain tissues after infusion of BTZ into the dBNST.

a Schematic diagram showing that BTZ was injected into the dBNST. Tissues from the dBNST, vBNST, IPACM, LSv, and CSF from LV were collected after infusion of BTZ into the dBNST. **b** The relative concentration of BTZ as quantified by LC-MS (n = 6). Error bars represent mean ± s.e.m. * $p < 0.05$, student t-test between the dBNST group vs each of the other four groups.

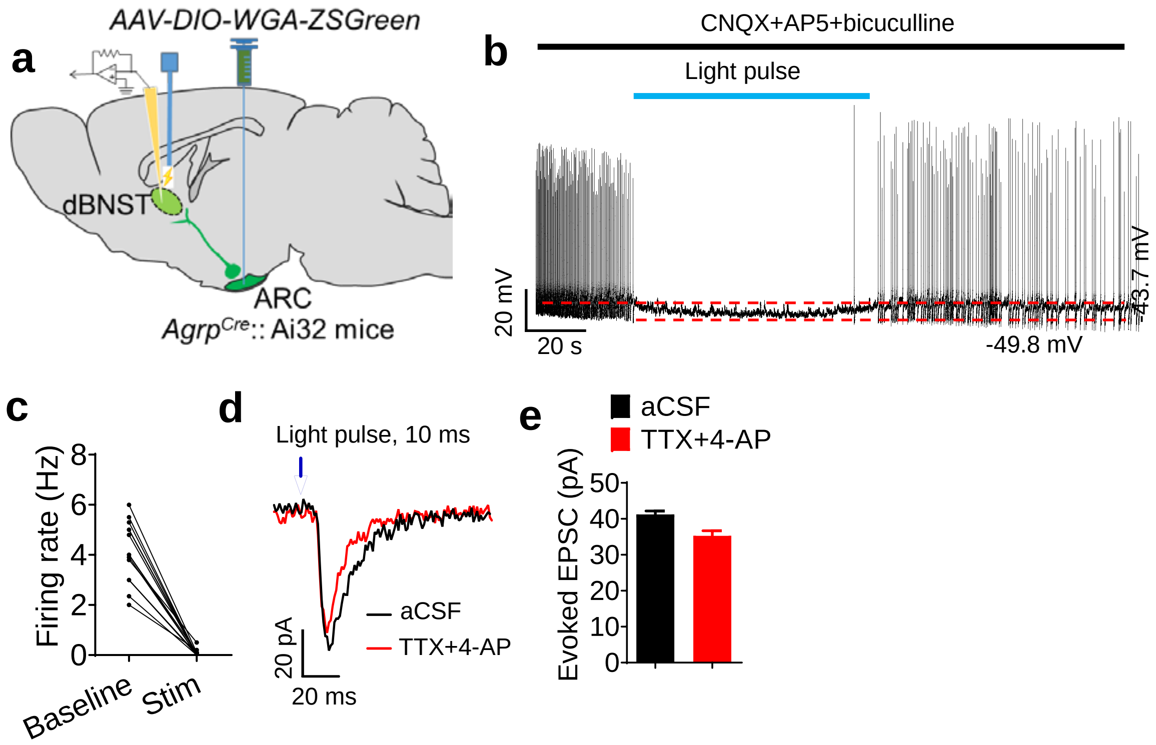

Extended Data Fig. 5. AgRP send direct projections to the MC4R neurons within the dBNST. **a** Schematic illustration of patch clamp recording from ZsGreen-expressing dlDRN neurons in *Agrp*^{Cre}*::*Ai32 mice with bilateral injection of *AAV9-DIO-WGA-ZsGreen* into the ARC*.* **b** Representative spikes of ZsGreen-labeled dBNST neurons before and after blue light pulses (10 ms/pulse, 20 Hz) shined onto AgRP axonal fibers. **c** Firing frequency analysis of neurons recorded in **b**. **d** The response of EPSCs recorded from a Mc4r^{dBNST} neuron upon photostimulation of AgRP^{dBNST} terminals in dBNST (10 ms pulse) with aCSF or TTX+4-AP. **e** Statistical analysis of light-evoked EPSC amplitudes of mice described in **d**. $p > 0.05$, student t-test between the aCSF group and the TTX+4-AP group.

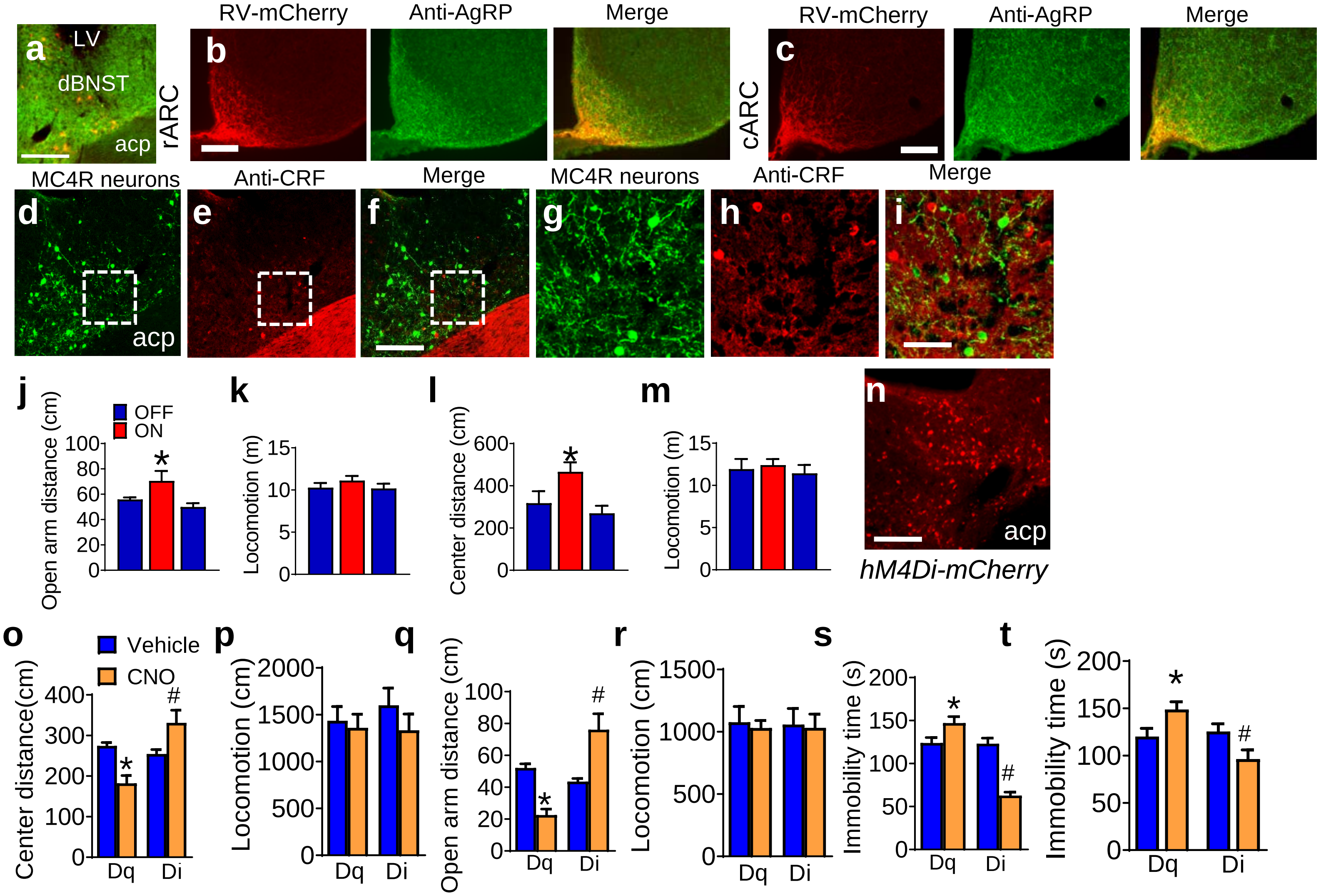

Extended Data Fig. 6. The ARC^{AgRP}-dBNST^{MC4R} circuit mediates anxiety and depression.

a, *AAV9-DIO-GTB* virus was injected into the dBNST of *Mc4^{Cre}* mice followed by injections of retrograding *EnvA-G-delete Rabies-mCherry* into the dBNST. Scale bar, 200 µm. **b** and **c**, Rabies-mCherry expression and anti-AgRP within the dorsal (**b**) and rostral ARC (**c**) . Scale bar in **b**, 30 µm; scale bar in **c**, 30 µm. **d**-**i**, Co-localization of MC4R^{dBNST} neurons with CRF. **d**-**f**, The immunostaining of CRF in the dBNST of the *Mc4r^{Cre}* mice with *AAV9-DIO-GFP* injection into dBNST. **g**-**i** is magnified view of the boxed area in **d**-**f**. Scale bar in **f** for **d**-**f**, 150 µm; scale bar in **i** for **g**-**i**, 50 µm. **j**, Distance travelled in the open arm. **k**, Total distance travelled in EPM. **l**, Distance travelled in the center zone. **m**, Total distance travelled in the open field of *Agrp^{Cre}* mice and *Agrp^{Cre}*::Ai32 mice upon optical stimulation in the dBNST. Error bars represent mean ± SEM. n = 7 per group; *$p < 0.05$; two-way ANOVA and followed by Bonferroni comparisons test. **n**, Viral transduction pattern of *Mc4r^{Cre}* mice with injection of *AAV9-FLEX-Hm4Di-mCherry* into the dBNST. **o-t**, OFT (**o, p**), epm (**q, r**), FST (**s**), and TST (**t**) test by *Mc4r^{Cre}* mice with injection of *AAV9-FLEX-hM3Dq-mCherry or AAV9-FLEX-hM4Di-mCherry* into the dBNST and treated with or without CNO. Error bars represent mean ± SEM. n = 7 per group; *$p < 0.05$; two-way ANOVA and followed by Bonferroni comparisons test.

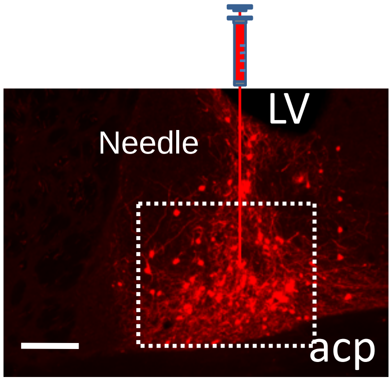

Extended Data Fig. 7. Viral injection within the dBNST.
Mc4r^{Cre} mice were injected with *AAV9-FLEX-hM3Dq-mCherry* into the dBNST and the viral expression was verified 4 weeks later. Scale bar, 200 μm.

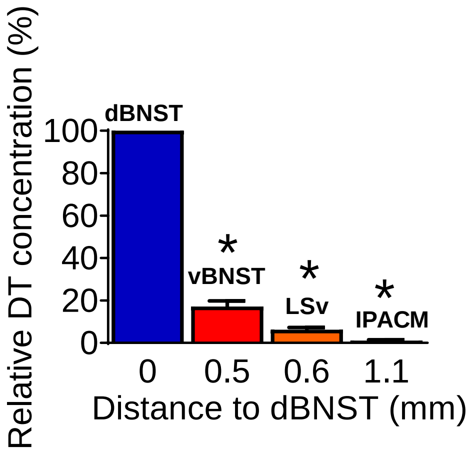

Extended Data Fig. 8. Quantification of the diffusion in brain tissues after infusion of DT into the dBNST.

The relative DT concentration was quantified by LC-MS in the dBNST, vBNST, LSv, and IPACM after infusion of DT into the dBNST of *Agrp*^{DTR} *mice* (n = 6) . Error bars represent mean ± s.e.m. * $p < 0.05$, student t-test between the dBNST group vs each of the other four groups.

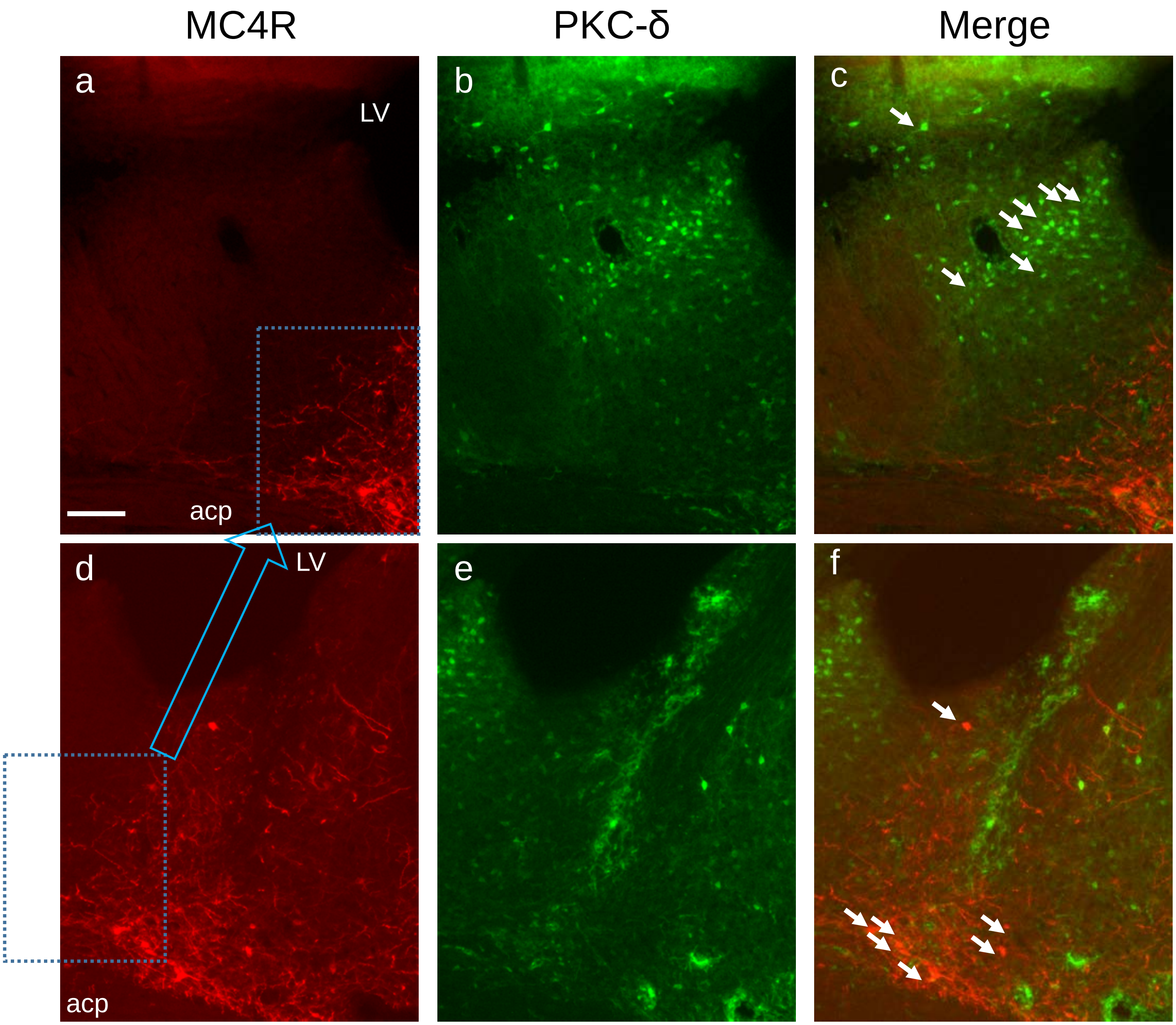

Extended Data Fig. 9. Expression profile of PKC-δ and MC4R wthin the dBNST.
a-c Anti-PKC-δ staining within dBNST (medial view) of *Mc4r*^{Cre}*::Rosa26*^{tdTomato} mice showed that PKC-δ is predominately expressed within the medial part of dBNST, whereas *MC4R* neurons are located mainly within the lateral part of dBNST. **d-f** Anti-PKC-δ staining within dBNST (lateral view) of the mice as described in a-c. Scale bar, 200 mm. White arrows showing the not overlapping *MC4R* neurons and PKC-δ neurons in **c** and **f**, respectively. Scale bar, 200 μm.

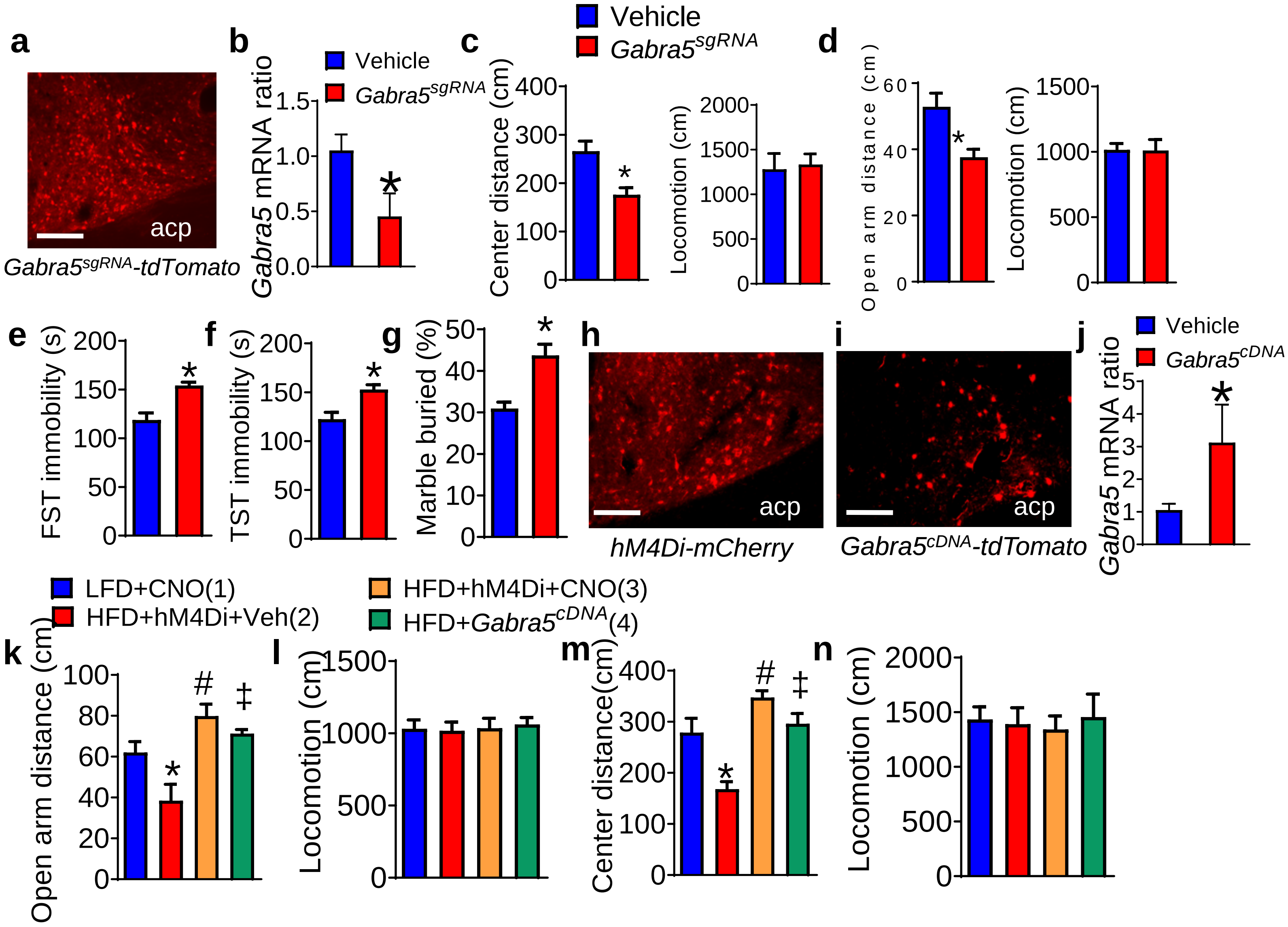

Extended Data Fig. 10. GABA$_A$-α5 within the MC4R^{dBNST} neurons regulates HFD-induced mental dysfunction and obesity.

a, Viral transduction pattern of *Mc4r^{Cre}::Rosa26$^{Cas9\text{-}EGFP}$* mice with injection of *AAV9-FLEX-Gabra5^{sgRNA}-tdTomato* into the dBNST. Scale bar in **a**, 150 μm. **b**, Real-time qPCR analysis of transcript levels of *Gabra5* expressed in MC4R neurons from the mice described in **a**. Error bars represent mean ± SEM. n = 6-8 per group; *$p < 0.05$; one-way ANOVA and followed by Tukey comparisons test. **c**-**g**, **c**, Distance travelled in the center of open field (left) and total distance travelled in the open field are examined (right). **d**, Distance travelled in the open arm (left) and total distance travelled (right) in EPM. Immobility time in the FST (**e**), TST (**f**) and percentage of the number of buried marbles in MBT assay (**g**) by *Mc4r^{Cre}::Rosa26$^{Cas9\text{-}EGFP}$* mice with injection of *AAV9-FLEX-Gabra5^{sgRNA}-tdTomato* injection (*Gabra5^{sgRNA}*) or *AAV9-FLEX-tdTomato* (Control) into the dBNST. Error bars represent mean ± SEM. n = 7-10 per group; *$p < 0.05$; one-way ANOVA and followed by Tukey comparisons test. **h**, Viral transduction pattern of *Mc4r^{Cre}* mice with injection of *AAV9-FLEX-hM4Di-mCherry* into the dBNST. Scale bar in **h**, 100 μm. **i**, Viral transduction pattern of *Mc4r^{Cre}* mice with injection of *AAV9-FLEX-Gabra5^{cDNA}- tdTomato* into the dBNST. Scale bar in **i**, 100 μm. **j**, Real-time qPCR analysis of transcript levels of *Gabra5* expressed in MC4R^{dBNST} neurons from the mice described in **i**. Error bars represent mean ± SEM. n = 6-9 per group; *$p < 0.05$; one-way ANOVA and followed by Tukey comparisons test. **k**-**n**, EPM (**k** and **l**) and OFT (**m** and **n**) test in CNO or vehicle-treated *Mc4r^{Cre}* mice with either *AAV9-FLEX-hM4Di-mCherry* or *AAV9-FLEX-Gabra5^{cDNA}- tdTomato* into the dBNST followed with chronic treatment of HFD or LFD. Error bars represent mean ± SEM. n = 8 per group; *$p < 0.05$, LFD+CNO vs HFD+hM4Di+Veh; $^{\#}p > 0.05$, LFD+CNO vs HFD+hM4Di+CNO; $^{\ddagger}p > 0.05$, LFD+CNO vs HFD+*Gabra5^{cDNA}*; one-way ANOVA and followed by Tukey comparisons test.

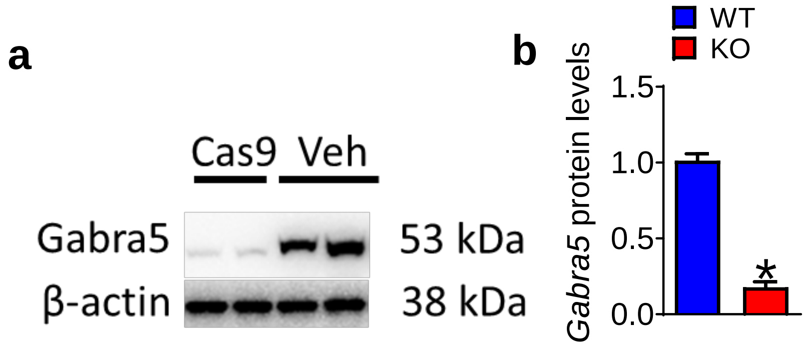

Extended Data Fig. 11. Western blot analysis of *Gabra5* expression within the dBNST.
a and **b** Western blotting (**a**) with quantification results (**b**) showing expression level of *Gabra5* with relative to β-actin from the dBNST of *Mc4r*^{Cre}*::Rosa26*$^{Cas9\text{-}EGFP}$ mice after injection of *AAV9-FLEX-Gabra5*^{sgRNA}*-tdTomato* into the dBNST (*n*=5-7 mice/group). Bars and error bars depict the mean ± SEM. * $p < 0.05$, student t-test between the dBNST group vs each of the other four groups.

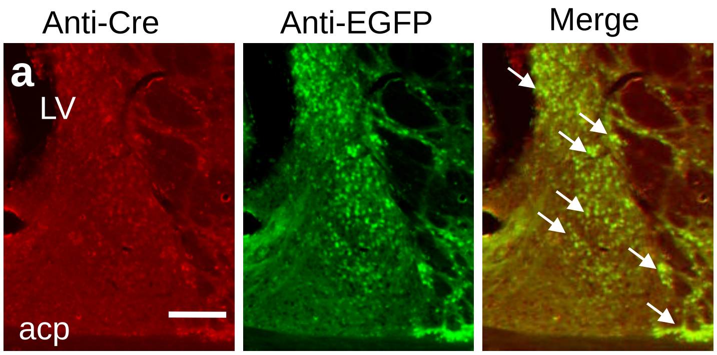

Extended Data Fig. 12. Expression profile of Cas9-EGFP within the MC4R^{dBNST} neurons.
a Representative immunofluorescence images of the dBNST from *Mc4r*^{Cre}*::Rosa26*$^{Cas9\text{-}EGFP}$ mice showing expression of Cas9-EGFP (anti-EGFP) is restricted to Cre-expressing neurons (anti-Cre). Scale bar, 150 μm.

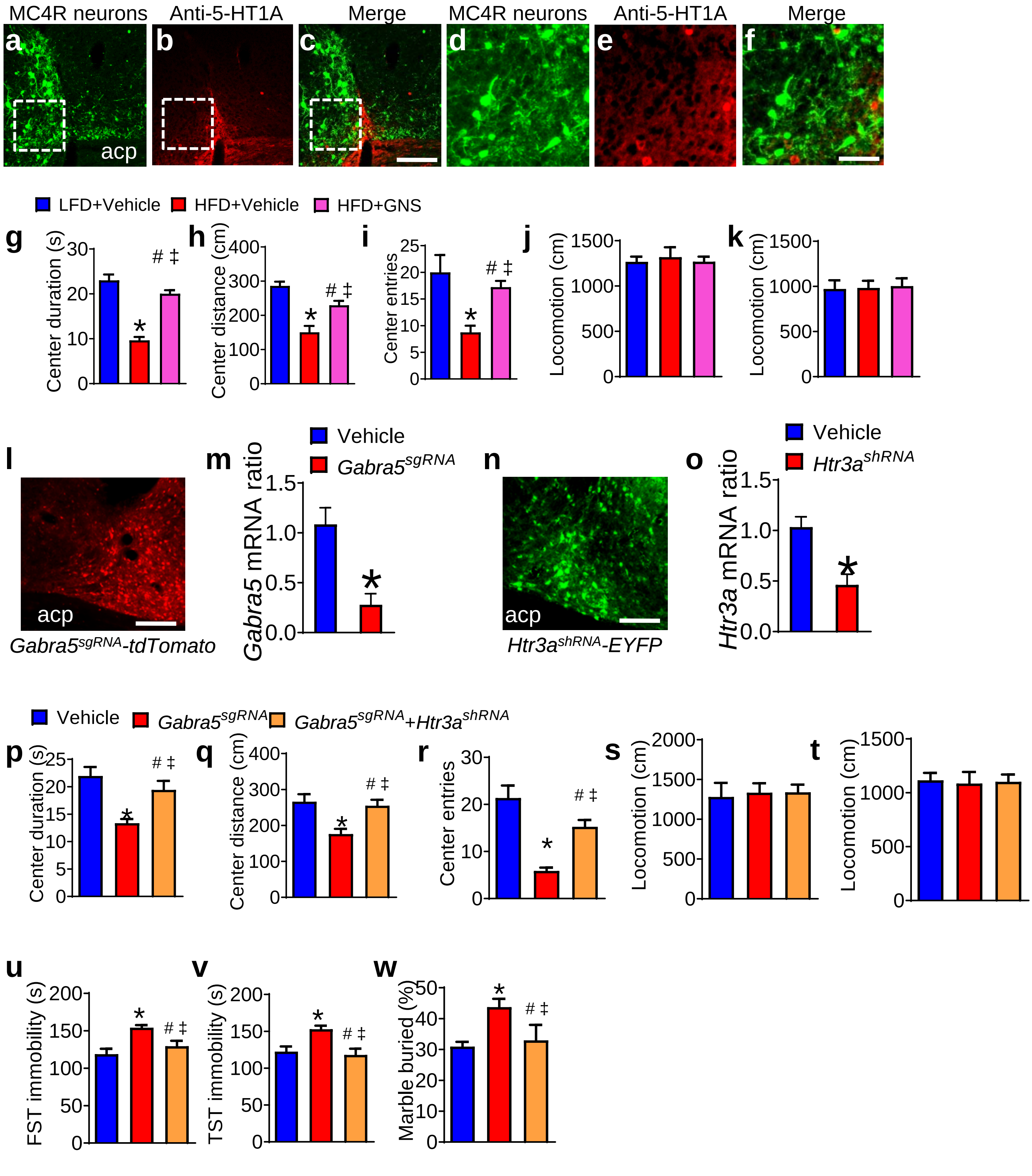

Supplementary Figure 13

Extended Data Fig. 13. Synergistic effect of the 5-HT$_3$R and GABA$_A$R-α5 signaling within the MC4R^{dBNST} neurons on HFD-induced mental deficiency and obesity.

a-**f**, Co-localization of MC4R^{dBNST} neurons with 5-HT$_1$A. **a**-**c**, The immunostaining of 5-HT1A in the dBNST of the *Mc4r^{Cre}* mice with *AAV9-DIO-GFP* injection into dBNST. **d**-**f,** magnified view of the boxed area in **a**-**c**. Scale bar in **c** for **a**-**c**, 150 μm; scale bar in **f** for **d**-**f**, 50 μm. **g**-**k**, The OFT (**g**-**j**) and EPM test (**k**) were performed in GNS or vehicle-treated mice with chronic treatment of HFD or LFD. Error bars represent mean ± SEM. n = 7-9 per group; *p < 0.05, LFD+Vehicle vs HFD+Vehicle; $^{\#}$p < 0.05, HFD+Vehicle vs HFD+GNS; ‡p > 0.05, LFD+Vehicle vs HFD+GNS. one-way ANOVA and followed by Tukey comparisons test. **l**, Viral transduction pattern of *Mc4r^{Cre}::Rosa26$^{Cas9-EGFP}$* mice with injection of *AAV9-FLEX-Gabra5^{sgRNA}-tdTomato* into the dBNST. Scale bar in **l**, 150 μm. **m**, Real-time qPCR analysis of transcript levels of *Gabra5* expressed in MC4R^{dBNST} neurons from the mice described in **l**. Error bars represent mean ± SEM. n = 6-8 per group; *p < 0.05; one-way ANOVA and followed by Tukey comparisons test. **n**, Viral transduction pattern of *Mc4r^{Cre}* mice with injection of *AAV9-FLEX-Htr3a^{shRNA}-EYFP* into the dBNST. Scale bar in **n**, 150 μm. **o**, Real-time qPCR analysis of transcript levels of *Htr3a* expressed in MC4R^{dBNST} neurons from the mice described in **n**. Error bars represent mean ± SEM. n = 6-7 per group; *p < 0.05; one-way ANOVA and followed by Tukey comparisons test. **p**-**w**, OFT (**p**-**s**), EPM (**t**), FST (**u**), TST (**v**) and MBT (**w**) by *Mc4r^{Cre}::Rosa26$^{Cas9-EGFP}$* mice with injection of either vehicle (Vehicle), *AAV9-FLEX-Gabra5^{sgRNA}-tdTomato* (Gabra5^{sgRNA}), or *AAV9-FLEX-Gabra5^{sgRNA}-tdTomato* and *AAV9-FLEX-Htr3a^{shRNA}-EYFP* (Gabra5^{sgRNA} + Htr3a^{shRNA}) within the dBNST. Error bars represent mean ± SEM. n = 6-8 per group; *p < 0.05, Vehicle vs Gabra5^{sgRNA}; $^{\#}$p < 0.05, Gabra5^{sgRNA} vs Gabra5^{sgRNA} + Htr3a^{shRNA}; ‡p > 0.05, Vehicle vs Gabra5^{sgRNA} + Htr3a^{shRNA}; one-way ANOVA and followed by Tukey comparisons test.

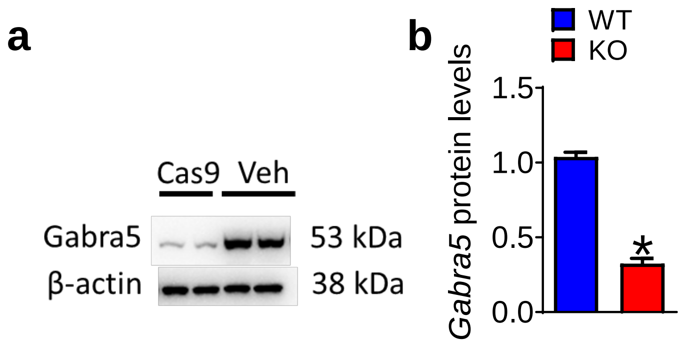

Extended Data Fig. 14. Western blot analysis of *Gabra5* expression within the dBNST.
a and **b** Western blotting (**a**) with quantification results (**b**) showing expression level of *Gabra5* with relative to β-actin from the dBNST of *Mc4r*^{Cre}*::Rosa26*$^{Cas9\text{-}EGFP}$ mice after injection of *AAV9-FLEX-Gabra5*^{sgRNA}*-tdTomato* into the dBNST (*n*=5-7 mice/group). Bars and error bars depict the mean ± SEM. * $p < 0.05$, student t-test between the dBNST group vs each of the other four groups.

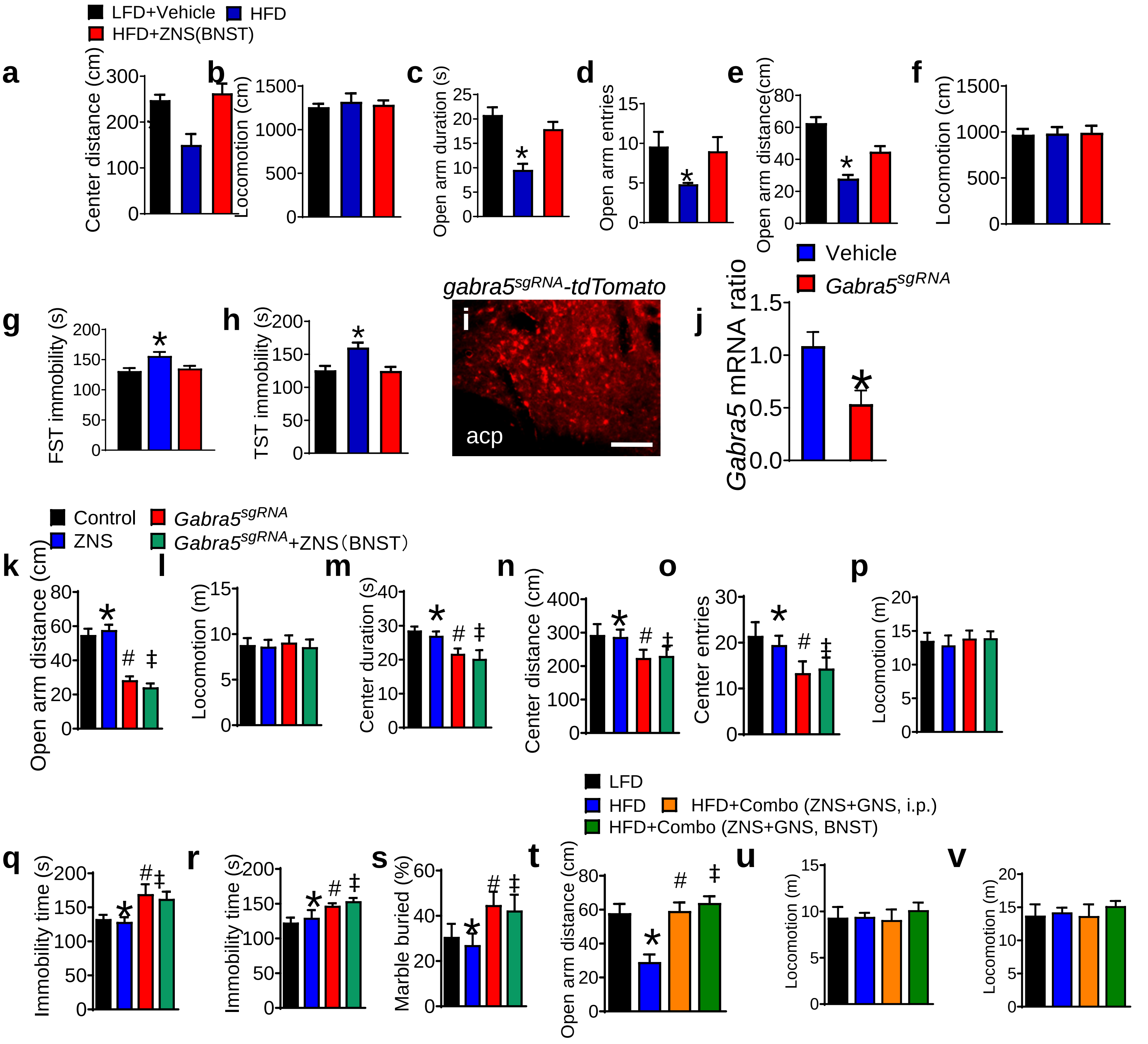
LFD+Vehicle
HFD
HFD+ZNS(BNST)
a
b
c
d
e
f
Center distance (cm)
Locomotion (cm)
Open arm duration (s)
Open arm entries
Open arm distance(cm)
Locomotion (cm)
Vehicle
Gabra5sgRNA
g
h
i
j
FST immobility (s)
TST immobility (s)
gabra5sgRNA-tdTomato
acp
Gabra5 mRNA ratio
Control
ZNS
Gabra5sgRNA
Gabra5sgRNA+ZNS（BNST）
k
l
m
n
o
p
Open arm distance (cm)
Locomotion (m)
Center duration (s)
Center distance (cm)
Center entries
Locomotion (m)
LFD
HFD
HFD+Combo (ZNS+GNS, i.p.)
HFD+Combo (ZNS+GNS, BNST)
q
r
s
t
u
v
Immobility time (s)
Immobility time (s)
Marble buried (%)
Open arm distance (cm)
Locomotion (m)
Locomotion (m)

Extended Data Fig. 15. Combination of zonisamide and granisetron alleviates HFD-induced mental dysfunctions and rescues obesity.

a-**h**, Behavior test of ad-lib fed *Mcr4^{Cre}* mice with *AAV9-FLEX-GCaMP6f* into the dBNST followed with chronic treatment of control LFD or HFD or HFD combined with i.c.v injection of ZNS. EPM (**a**, **b**), OFT (**c**-**f**), FST (**g**), and TST (**h**) assays. Error bars represent mean ± SEM. n = 7-9 per group; *p < 0.05, LFD vs HFD; $^{\#}$p > 0.05, HFD+ZNS vs LFD. One-way ANOVA and followed by Tukey comparisons test. **i**, Viral transduction pattern of *Mc4r^{Cre}::Rosa26$^{Cas9\text{-}EGFP}$* mice with injection of *AAV9-FLEX-Gabra5^{sgRNA}-tdTomato* into the dBNST. Scale bar in **i**, 150 μm. **j**, Real-time qPCR analysis of transcript levels of *Gabra5* expressed in MC4R neurons from the mice described in **j**. Error bars represent mean ± SEM. n = 7-8 per group; *p < 0.05; one-way ANOVA and followed by Tukey comparisons test. **k**-**s**, Behavior test of in ad-lib fed *Mc4r^{Cre}::Rosa26^{Cas9}* mice with bilateral injection of either vehicle, *AAV9-FLEX-Gabra5^{sgRNA}-tdTomato* (Gabra5^{sgRNA}) into dBNST, followed with microinjection of vehicle or ZNS into dBNST. EPM (**k**, **l**), OFT (**m**-**p**), FST (**q**), TST (**r**), and MBT (**s**) assays. Error bars represent mean ± SEM. n = 7-9 per group; *p < 0.05, Vehicle vs Gabra5^{sgRNA}; $^{\#}$p < 0.05, Vehicle vs ZNS; ‡p > 0.05, Gabra5^{sgRNA} vs Gabra5^{sgRNA}+ZNS. One-way ANOVA and followed by Tukey comparisons test. **t**-**v,** Behavior test of chronic treatment of control LFD or HFD or HFD combined with i.p. injection of ZNS and GNS into dBNST or HFD combined with microinjection of ZNS and GNS into dBNST on WT mice. EPM (**t** and **u**) and OFT (**v**) assays. Error bars represent mean ± SEM. n = 7 per group; *p < 0.05, HFD vs HFD+ZNS+GNS (i.p.); $^{\#}$p < 0.05, HFD vs HFD+ZNS+GNS (BNST); two-way ANOVA and followed by Bonferroni comparisons test.
